# Histone demethylase LSD1 restricts influenza A virus infection by erasing IFITM3-K88 monomethylation

**DOI:** 10.1371/journal.ppat.1006773

**Published:** 2017-12-27

**Authors:** Jiaoyu Shan, Binbin Zhao, Zhao Shan, Jia Nie, Rong Deng, Rui Xiong, Andy Tsun, Weiqi Pan, Hanzhi Zhao, Ling Chen, Ying Jin, Zhikang Qian, Kawing Lui, Rui Liang, Dan Li, Bing Sun, Dimitri Lavillette, Ke Xu, Bin Li

**Affiliations:** 1 CAS Center for Excellence in Molecular Cell Science, CAS Key Laboratory of Molecular Virology and Immunology, Unit of Molecular Immunology, Institut Pasteur of Shanghai, Shanghai Institutes for Biological Sciences, Chinese Academy of Sciences, Shanghai, China; 2 Human Parasitology Department of Basic Medicine College, Xinjiang Medical University, Urumqi, China; 3 Shanghai Institute of Immunology, Shanghai JiaoTong University School of Medicine, Shanghai, China; 4 Department of Immunology and Microbiology, Shanghai JiaoTong University School of Medicine, Shanghai JiaoTong University, Shanghai, China; 5 CAS Key Laboratory of Molecular Virology and Immunology, Unit of interspecies transmission of arboviruses and therapeutics research, Institut Pasteur of Shanghai, Shanghai Institutes for Biological Sciences, Chinese Academy of Sciences, Shanghai, China; 6 Guangzhou Institutes of Biomedicine and Health, Chinese Academy of Sciences, Guangzhou, China; 7 CAS Center for Excellence in Molecular Cell Science, Key Laboratory of Stem Cell Biology, Institute of Health Sciences, Shanghai Institutes for Biological Sciences, Chinese Academy of Sciences/Shanghai JiaoTong University School of Medicine, Shanghai, China; 8 CAS Key Laboratory of Molecular Virology and Immunology, Unit of Herpesvirus and Molecular Virology Research, Institut Pasteur of Shanghai, Shanghai Institutes for Biological Sciences, Chinese Academy of Sciences, Shanghai, China; 9 CAS Center for Excellence in Molecular Cell Science, CAS Key Laboratory of Molecular Virology and Immunology, Unit of Molecular Virology, Institut Pasteur of Shanghai, Shanghai Institutes for Biological Sciences, Chinese Academy of Sciences, Shanghai, China; UNITED STATES

## Abstract

The histone demethylase LSD1 has been known as a key transcriptional coactivator for DNA viruses such as herpes virus. Inhibition of LSD1 was found to block viral genome transcription and lytic replication of DNA viruses. However, RNA virus genomes do not rely on chromatin structure and histone association, and the role of demethylase activity of LSD1 in RNA virus infections is not anticipated. Here, we identify that, contrary to its role in enhancing DNA virus replication, LSD1 limits RNA virus replication by demethylating and activating IFITM3 which is a host restriction factor for many RNA viruses. We have found that LSD1 is recruited to demethylate IFITM3 at position K88 under IFNα treatment. However, infection by either Vesicular Stomatitis Virus (VSV) or Influenza A Virus (IAV) triggers methylation of IFITM3 by promoting its disassociation from LSD1. Accordingly, inhibition of the enzymatic activity of LSD1 by Trans-2-phenylcyclopropylamine hydrochloride (TCP) increases IFITM3 monomethylation which leads to more severe disease outcomes in IAV-infected mice. In summary, our findings highlight the opposite role of LSD1 in fighting RNA viruses comparing to DNA viruses infection. Our data suggest that the demethylation of IFITM3 by LSD1 is beneficial for the host to fight against RNA virus infection.

## Author summary

The viral genomes of DNA viruses but not RNA viruses form chromatin structure during infection. Thus, epigenetic modulators are not expected to have crucial roles in RNA viral infection. However, here, we identify for the first time, that, opposite to its role in enhancing DNA virus replication, LSD1, a histone demethylase, limits RNA virus replication. We show that, under IFNα treatment, LSD1 is involved in the demethylation of IFITM3, a well-known host restriction factor for many RNA viruses. To counteract IFITM3 activation by demethylation, several RNA viruses, such as VSV and IAV, but not Zika virus, have developed strategy to inactive IFITM3 by promoting its dissociation from LSD1. In agreement with our findings, the inhibition of the enzymatic activity of LSD1 by small molecule leads to more severe disease outcomes in IAV-infected mice. Our data suggest that although LSD1 inhibitor is beneficial for treating DNA virus infection, it could be harmful to the host suffering from RNA virus infection. On the contrary, developing strategies to stimulate LSD1 activity to demethylate of IFITM3 is essential to fight RNA viruses.

## Introduction

DNA virus genomes are encapsidated without histones, but rapidly acquire chromatin structure upon infection[1, 2]. Thus, epigenetic regulators, such as histone methylase and demethylase, are found to be able to regulate DNA virus replication. Recently, increasing studies have shown that inhibition of LSD1 (lysine-specific demethylase 1; KDM1A family), a histone demethylase, can block viral genome transcription and lytic replication of DNA viruses. For herpes virus, inhibition of LSD1 suppresses viral genome transcription and lytic replication, so that virus shedding and disease severity were reduced[3–5]. LSD1 is also found to activate HBV transcription by establishing an active hepatitis B viral chromatin state[6]. Moreover, inhibition of LSD1 activity suppresses the activation of HIV transcription in latently infected T cells by demethylation of viral Tat protein[7]. However, different from DNA viruses, RNA viruses do not rely on chromatin structure and histone capsid. As a result, the activity of LSD1 is expected to have little effect on RNA virus genome transcription via histone demethylation. Nevertheless, it is still possible that LSD1 influences RNA virus replication through demethylation of other host or viral proteins.

The interferon-inducible transmembrane (*IFITM*) gene family, belonging to a group of small interferon stimulated genes (ISGs)[8], has recently been identified to exhibit antiviral activities to a broad spectrum of viruses through blocking the early stages of viral life cycle[9, 10]. In humans, there are at least four functional members of IFITM proteins. IFITM1, 2 and 3 are expressed in a variety of human tissues and cell lines. IFITM5 is limited to the bone and is involved in mineralization[11]. Among these IFITM proteins, IFITM3, located mainly in endosome membrane, is well characterized for the restriction of RNA viruses such as IAV [12, 13]. IFITM3 protects the mice against morbidity and mortality due to influenza virus infection[12, 14], and in humans, disease severity in IAV-infected patients are correlated with the C allele (SNP rs12252) of human *IFITM3*[15]. Recently, our work showed that IFITM3 is mono-methylated on lysine residue of K88 in conserved intercellular loop (CIL) domain to reduce its antiviral activity[16, 17]. We therefore search for the corresponding demethylase to stimulate IFITM3 activity.

IFITMs reside in the cytosolic leaflet of plasma or endosome/lysosome membranes with the help of two intermembrane domains (IM1 and IM2)[18, 19]. Between IM1 and IM2 is the CIL domain, and on side of IM1 and IM2 are the cytosolic N-terminus and C-terminus respectively[19, 20]. The CIL domain is exposed to cytosol and is intensively regulated by post-translational modifications. The conserved cysteine residues in CIL domain were reported to be palmitoylated, which is required for protein clustering of IFITMs on the membrane and its antiviral function[21]. Ubiquitination on any of the four lysines in the N-terminus and CIL of IFITM3 negatively regulated its antiviral activity by accelerating degradation of IFITM3[22]. Through mass spectrometry assay, we have identified a monomethylation site at K88 located in CIL domain of IFITM3. Methylation of IFITM3 is catalyzed by SET7 to inactivate antiviral activities of IFITM3[16].

In this study, the lysine specific demethylase (LSD1) is identified to demethylate the K88me1 modification of IFITM3. The interaction strength between LSD1 and IFITM3 positively correlates with the antiviral activity of IFITM3. We further verified that IFNα treatment triggers demethylation of IFITM3 by LSD1 to be antiviral active. We showed that RNA viruses like IAV and VSV have developed strategies to counteract IFITM3 by disassociating LSD1 from IFITM3. However, surprisingly, Zika virus, which may not yet well adapt to human, could not counteract IFITM3 by methylation. Unlike herpes-virus infected animals, inhibition of LSD1 in IAV-infected mice aggravates the disease severity and increases the levels of IFITM3-K88me1, suggesting that LSD1 act differently in DNA and RNA virus infections. The action of LSD1 to demethylate IFITM3 is essential for the host to fight against RNA viruses.

## Results

### IFNα recruits LSD1 to demethylate K88 of IFITM3

**Fig 1:**
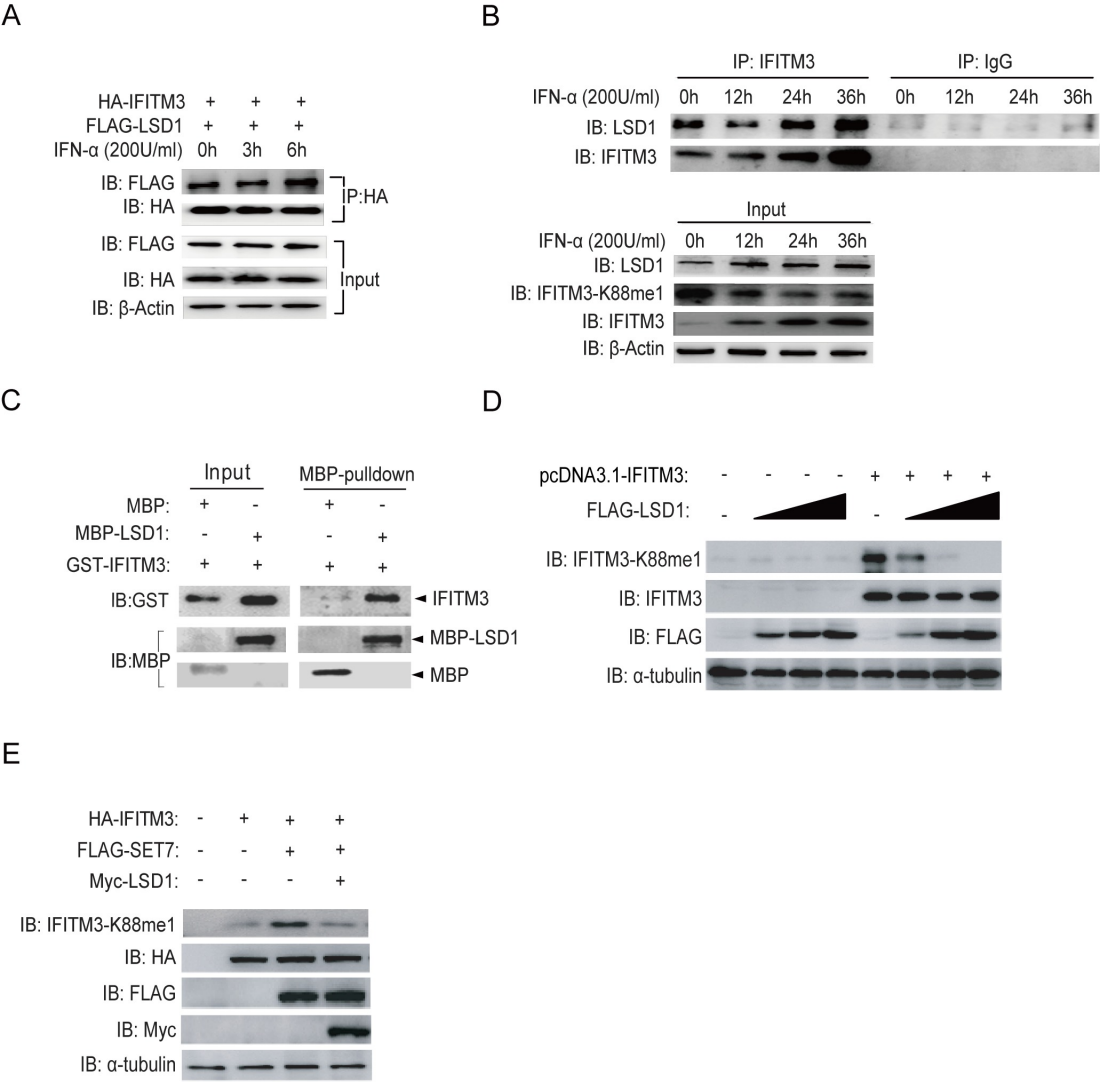
LSD1 catalyzes lysine demethylation of IFITM3 at K88. (A) HEK293T cells grown in six-well plates were transfected with HA-IFITM3 and FLAG-LSD1. Forty-eight hours later, cells were treated with IFNα (200U/ml) for the indicated time periods. IFITM3 was immunoprecipitation (IP) by anti-HA antibodies and LSD1 was probed with indicated antibody. The expression levels of HA-IFITM3, FLAG-LSD1 and β-Actin were shown as loading control (Input). (B) HEK293T cells grown in six-well plates were treated with IFNα (200U/ml) for the indicated time periods and were then collected to perform immunoprecipitation (IP) of endogenous IFITM3. The levels of LSD1 and IFITM3 were detected by immunoblotting with anti-LSD1 and anti-IFITM3 antibodies. Proteins levels in whole cell lysates were shown as Input. (C) The interaction of LSD1 and IFITM3 is direct. A pull down assay was performed with recombinant MBP-LSD1, MBP, and GST-IFITM3 as indicated. Immunoblotting (IB) was used to show protein levels. (D) LSD1 down-regulates monomethylation of IFITM3 at K88 in a dose dependent manner. HEK293T cells were transfected with pcDNA3.1-IFITM3 and increasing amounts of FLAG-LSD1 plasmids (0.6μg, 1.5μg or 2.4μg). Forty-eight hours later, cells were collected and levels of IFITM3-K88me1 were determined by western blotting. (E) The demethylase activity of LSD1 is antagonized with SET7. FLAG-tagged SET7, Myc-tagged LSD1 and HA-tagged IFITM3 were co-transfected into HEK293T cells as indicated. After 48h, the cells were collected and protein levels were detected by western blotting.

LSD1 was selected to test its demethylase activity on IFITM3, because it can antagonize SET7 for protein methylation[23]. We first tested whether LSD1 is recruited to IFITM3 under IFNα treatment. For this, HA-tagged IFITM3 and FLAG-tagged LSD1 were over-expressed in HEK293T cells followed by immunoprecipitation (IP) using anti-HA antibody to pull down IFITM3. As shown in Fig 1A, there is an intrinsic interaction between LSD1 and IFITM3 at 0h of IFNα treatment. Subsequently, clearly enhanced interaction between IFITM3 and LSD1 was detected at 6h post-IFNα-treatment. This increased interaction was also observed with endogenous IFITM3 and LSD1 upon IFNα treatment (Fig 1B), indicating that LSD1 responds to IFNα simulation via binding to IFITM3. As a consequence, the levels of IFITM3-K88me1 were reduced over time under IFNα treatment when more LSD1 binds to IFITM3 (Fig 1B, Input). Moreover, we confirmed that there was a physical interaction between IFITM3 and LSD1 *in vitro* by MBP pull-down assay (Fig 1C) which may explain the co-localization between LSD1 and IFITM3 has shown in S1 Fig.

To validate the demethylase activity of LSD1 on IFITM3, different doses of LSD1 were co-transfected with IFITM3 to examine the IFITM3-K88me1 levels. The data showed that LSD1 could demethylate IFITM3 at K88 in a dose-dependent manner (Fig 1D). Furthermore, the demethylase activity of LSD1 was antagonized with SET7 because IFITM3-K88me1 catalyzed by SET7 was largely reduced in the present of LSD1 (Fig 1E).

### LSD1 enhances the antiviral activity of IFITM3

**Fig 2:**
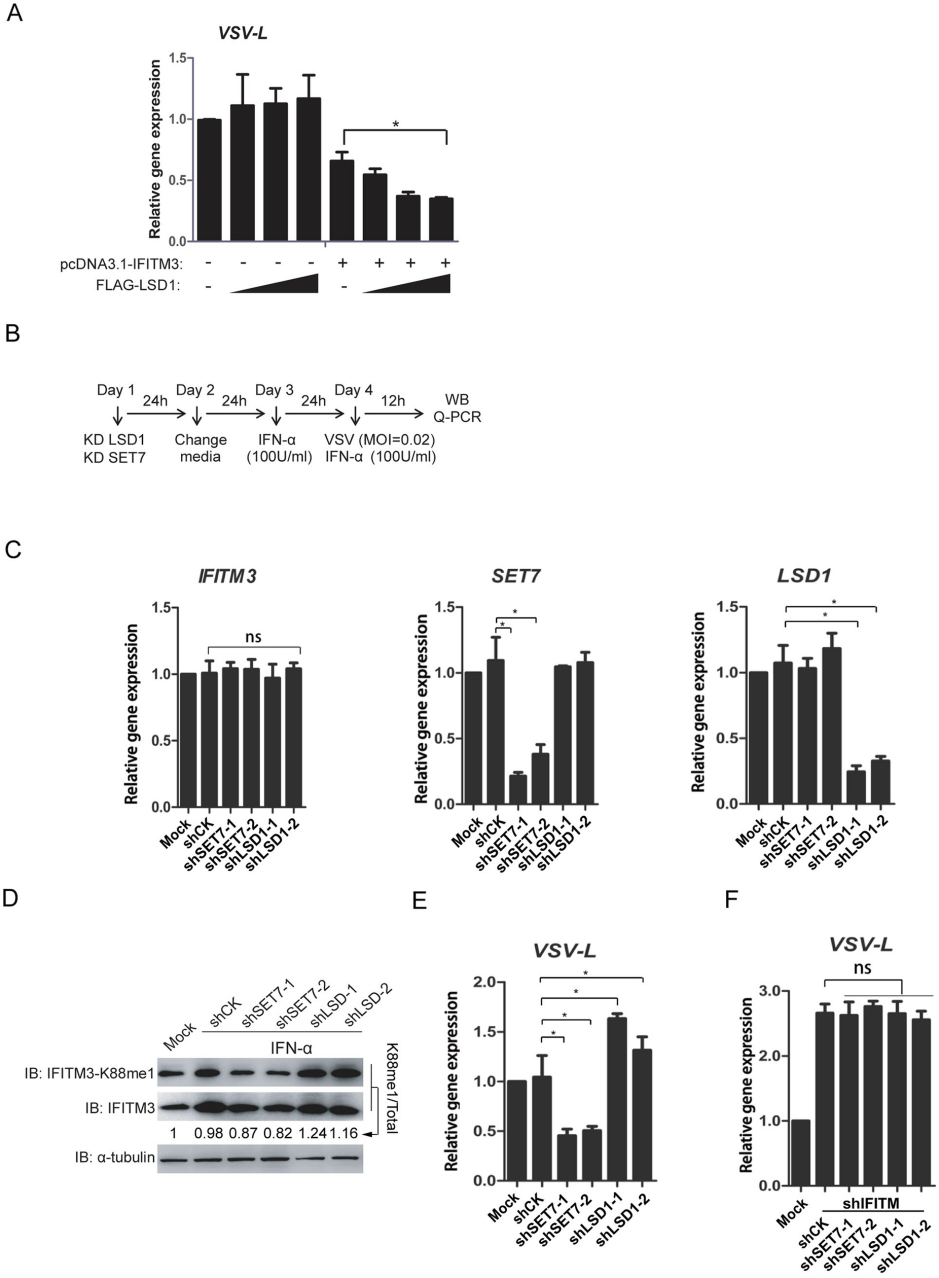
LSD1 enhances the antiviral activity of IFITM3 against VSV infection. (A) Overexpression of LSD1 promotes the antiviral activity of IFITM3. HEK293T cells were transfected with pcDNA3.1-IFITM3 and increasing amounts of FLAG-LSD1 plasmids (0.6μg, 1.5μg or 2.4μg). Forty-eight hours later, cells were infected with VSV at MOI = 0.02. The cells were then collected at 12h post-infection and tested by qPCR to detect of the vRNA levels of viral *L* gene. Experimental procedure is shown in B. (C) Knockdown efficiency of shRNAs against SET7 and LSD1. HEK293T cells were infected by lentivirus coding shRNA against either SET7 (sh*SET7*) or LSD1 (sh*LSD1*) followed by qPCR analyses of the mRNA levels of IFITM3, SET7 or LSD1 respectively. (D and E) Knockdown of LSD1 promotes the methylation of IFITM3 at K88, whereas, knockdown of SET7 reduces IFITM3-K88me1. Lentivirus-packaged shRNAs against either SET7 (sh*SET7*) or LSD1 (sh*LSD1*) were transduced into HEK293T cells. None-transduced 293T cells are included as control (Mock).The media was changed to fresh DMEM media 24h later. After another 24h, equal number of 5×10^5^ cells were transferred to twelve-well plates and then treated with IFNα (100U/ml) to induce the expression of IFITM3. Mock cells were treated without IFNα. Twenty-four hours after IFNα treatment. the cells were infected with VSV at MOI = 0.02 and were then collected at 12h post-infection for western blot to detect the levels of IFITM3 and IFITM3-K88me1 (D) or for qPCR to detect the vRNA levels of viral *L* gene (E). (F) The effects of LSD1 and SET7 are dependent on IFITM3 expression. Experimental procedure is same as procedure shown in B except that lentivirus-packaged shRNAs against either SET7 (sh*SET7*) and IFITM3 (sh*IFITM3*) or LSD1 (sh*LSD1*) and IFITM3 (sh*IFITM3*) were transduced into HEK293T cells on the Day 1. Cells were collected at 12h post-infection for qPCR to detect the vRNA levels of viral *L* gene. All data are representative of more than three independent experiments, and are shown by the mean value with +s.d. ns, *p* >0.05; *, *p* <0.05; **, *p* <0.01; ***, *p* <0.001.

To test the effect of LSD1 on IFITM3 activity towards virus infection, HEK293T cells were transfected with IFITM3 and LSD1 and were then infected with VSV. As expected, the replication of VSV (expressed by vRNA copies of VSV *L* gene) was distinctly reduced when LSD1 was co-expressed with IFITM3 in a dose-dependent manner (Fig 2A). To verify the effect of LSD1 on virus replication in a more physiological condition, HEK293T cells were transduced with lentivirus delivering control shRNA (shCK) or shRNA targeting either SET7 (sh*SET7*) or LSD1 (sh*LSD1*). After two days, cells were treated by IFNα (100U/ml) for additional 24h to induce endogenous expression of IFITM3, and were then infected with VSV. Cells were collected 12h post-infection, and the gene expressions of *IFITM3*, *LSD1*, and *SET7* as well as the levels of IFITM3-K88me1 were examined (Experimental procedure shown in Fig 2B). As expected, sh*SET7* and sh*LSD1* efficiently knocked down gene expression levels of *SET7* and *LSD1* respectively; while the expression of *IFITM3* was not affected (Fig 2C). The levels of IFITM3-K88me1 were up-regulated by sh*LSD1* but down-regulated by the sh*SET7* correspondingly (Fig 2D). Consisted with the levels of IFITM3-K88me1, sh*LSD1* caused augments in VSV vRNA levels while sh*SET7* remarkably reduced VSV vRNA levels (Fig 2E). To explore whether the effect of LSD1 and SET7 on VSV infection is dependent on IFITM3 activity, *IFITM3* and *LSD1* or *IFITM3* and *SET7* were knocked down together under the same experimental protocol (S2 Fig). The results in Fig 2F showed that, neither SET7 nor LSD1 had effects on VSV replication in the absence of IFITM3. The data implied that anti-viral activity of IFITM3 against VSV was dependent on the methylation levels of IFITM3-K88me1 and under the regulation of IFITM3 methylation modification enzymes of SET7 and LSD1.

**Fig 3:**
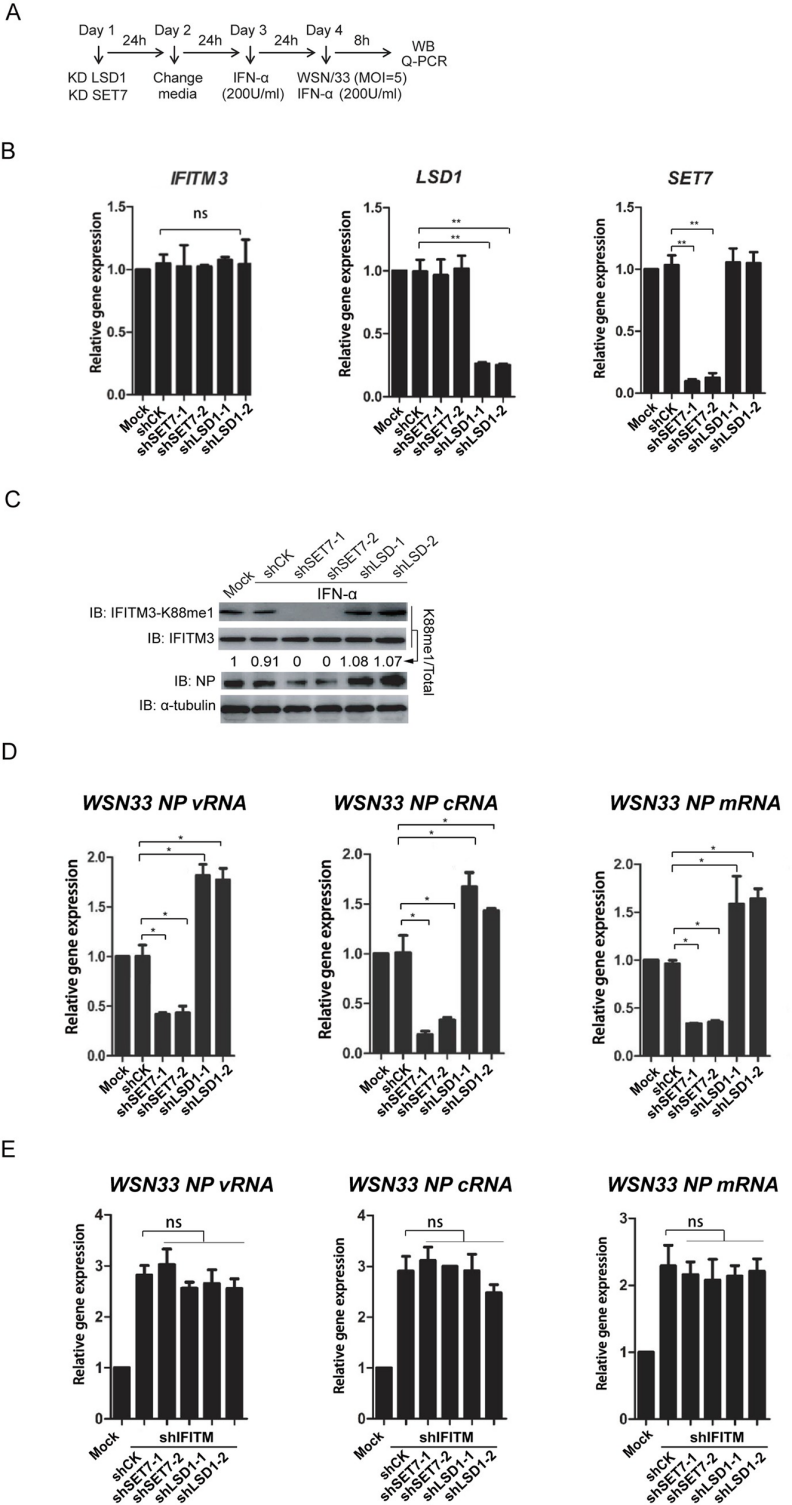
LSD1 enhances the antiviral activity of IFITM3 against IAV infection. Experimental procedure is shown in A. (B) Knockdown efficiency of shRNAs against SET7 and LSD1. HEK293T cells were infected by lentivirus coding shRNA against either SET7 (sh*SET7*) or LSD1 (sh*LSD1*) followed qPCR analyses of the mRNA levels of IFITM3, SET7 or LSD1 respectively. (C and D) Knockdown of LSD1 promotes the methylation of IFITM3 at K88, whereas, knockdown of SET7 reduces IFITM3-K88me1. Lentivirus-packaged shRNAs against either SET7 (sh*SET7*) or LSD1 (sh*LSD1*) were transduced into A549 cells. None-transduced A549 cells are included as control (Mock). The media was changed to fresh DMEM media 24h later. After another 24h, equal number of 5×10^5^ cells were transferred to twelve-well plates and then treated with IFNα (200U/ml) to induce the expression of IFITM3. Mock cells were treated without IFNα. Twenty-four hours after IFNα treatment, the cells were infected with WSN at MOI = 5 and were then collected at 8h post-infection for western blotting to detect the levels of IFITM3 and IFITM3-K88me1 (C) or for qPCR to detect the *vRNA*, *cRNA* and *mRNA* levels of viral gene (D). (E) The effects of LSD1 and SET7 are dependent of IFITM3 expression. Experimental procedure is same as procedure shown in A except that lentivirus-packaged shRNAs against either SET7 (sh*SET7*) and IFITM3 (sh*IFITM3*) or LSD1 (sh*LSD1*) and IFITM3 (sh*IFITM3*) were transduced into A549 cells on the Day 1. Cells were then collected at 8h post-infection for qPCR to detect the *vRNA*, *cRNA* and *mRNA* levels of viral gene. All data are representative of more than three independent experiments, and are shown by the mean value with +s.d. ns, *p* >0.05; *, *p* <0.05; **, *p* <0.01; ***, *p* <0.001.

To examine the broad-reactive activity of IFITM3 against virus infections, A549 cells were infected by influenza A virus, A/WSN/33 (H1N1) (WSN), with a similar experimental procedure (shown in Fig 3A). As expected, sh*SET7* and sh*LSD1* efficiently knocked down gene expression levels of *SET7* and *LSD1* respectively; while the expression of *IFITM3* was not be affected (Fig 3B). Transduction of sh*SET7* led to the reduced levels of IFITM3-K88me1, wherein there was also less expression of viral NP proteins (Fig 3C). By contrast, the increased levels of IFITM3-K88me1 by sh*LSD1* resulted in elevated expression of viral NP proteins (Fig 3D). The expressions of vRNA, cRNA and mRNA of viral segment 5 (NP) were suppressed by sh*SET7* but stimulated by sh*LSD1* (Fig 3D). Again, when *IFITM3* was knocked down together with *LSD1* or *SET7*, both SET7 and LSD1 lost their effects on influenza A virus replication (S3 Fig and Fig 3E).

Altogether, the data suggested that LSD1 positively regulated the antiviral activities of IFITM3 via down-regulating IFITM3-K88me1. On the contrary, SET7 negatively regulated the antiviral activities of IFITM3 via up-regulating IFITM3-K88me1.

### LSD1 inhibitor triggers severe disease progression in IAV-infected mice

**Fig 4:**
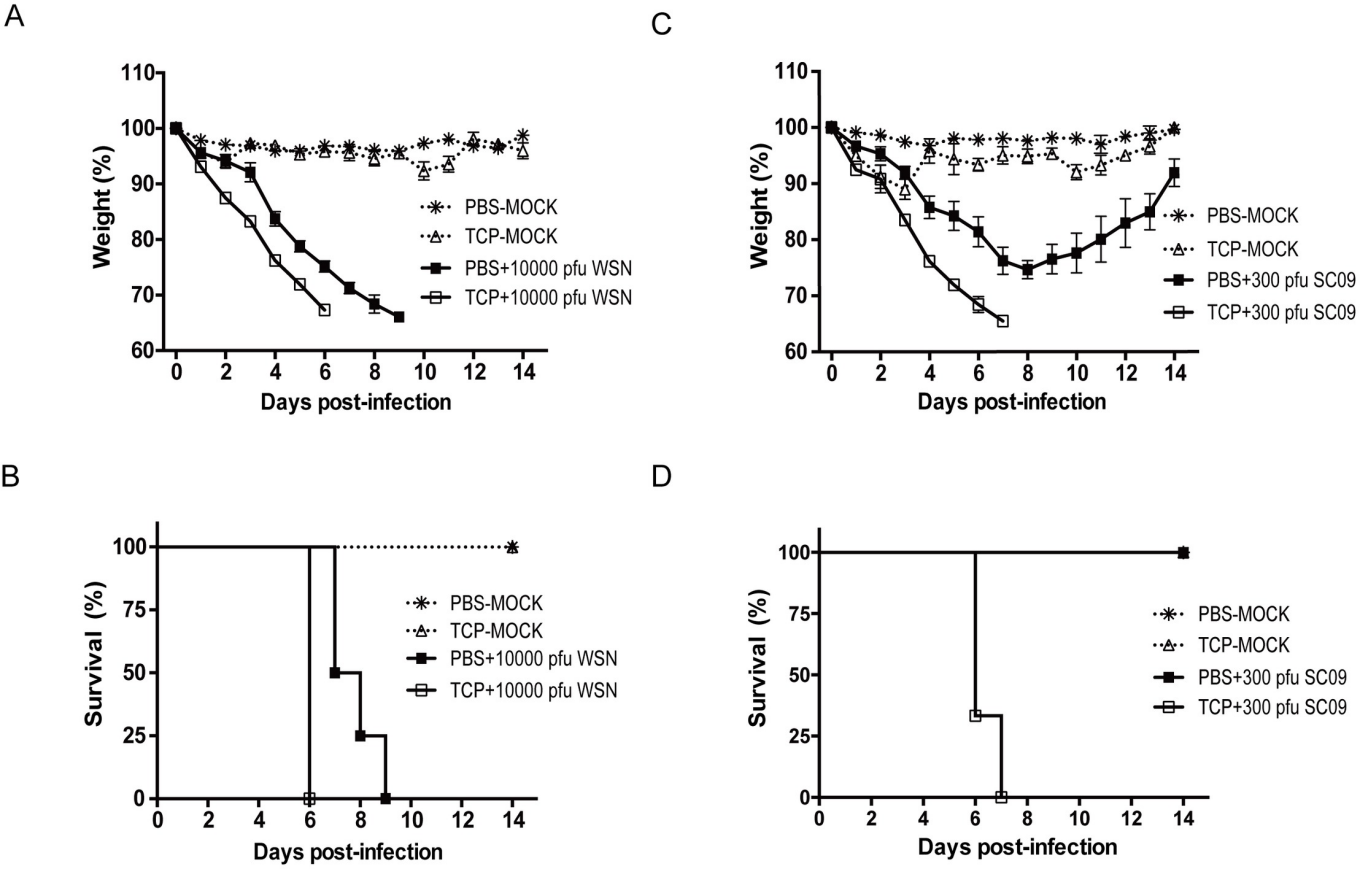
TCP treatment induces severe disease in IAV-infected mice. The mice (n = 4) were pretreated with PBS (100μl/kg) or TCP (5mg/kg) through intraperitoneally injection on day 0 (D0). One hour later, mice were infected with either 10000 pfu of WSN virus or 300 pfu of SC09 virus in 50μl PBS or 50μl PBS (mock) intranasally. All mice were injected intrapertoneally with PBS (100μl/kg) or TCP (5mg/kg) once a day. The body weights of mice were monitored throughout the infection time course from Day 0 to Day 14 (A and C). The survival curve of mice was shown in B and D. For mice infected with WSN virus, the differences between TCP-treatment and PBS-treatment groups were significant (p<0.05) from Day 2 to Day 6. For mice infected with SC09 virus, the differences between TCP-treatment and PBS-treatment groups were significant (p<0.05) from Day 3 to Day 6.

Currently, it remains unknown how IFITM3-K88me1 and its methylation enzymes affect disease outcome *in vivo*. By identifying the methyltransferase SET7 and the demethylase LSD1 of IFITM3, we were able to take the advantage of chemical inhibitors towards these enzymes to study the role of IFITM3-K88me1 in IAV-infected animals. A LSD1 inhibitor, Trans-2-phenylcyclopropylamine hydrochloride (TCP), was applied intraperitoneally to mock-infected or WSN-infected mice daily. A dose of 10000 pfu viruses were used to infect mice to guarantee a lethal infection. As shown in Fig 4A and Fig 4B, a mild body-weight loss was observed in TCP-treated mock group indicating limited toxicity of TCP. Compared with PBS-treatment, infected mice treated with TCP showed faster body weight loss (Fig 4A). The survival curve in Fig 4B clearly indicated that TCP treatment accelerated the death of IAV-infected mice. To examine the effect of TCP under the infection of a nature isolate, mice were infected again with A/Sichuan/1/2009 (H1N1) (SC09, a pandemic H1N1 stain) at a sublethal dose (300 pfu) followed by TCP or PBS treatment. The data in Fig 4C and Fig 4D showed that the PBS-treated infected mice recovered from body weight loss at day 8 and all survive, but none of the TCP-treated infected mice could survive more than 7 days. However, compared with wild-type mice, it was worth noting that there was no effect of TCP in IFITM-knockout mice in IAV infection (S4 Fig).

**Fig 5:**
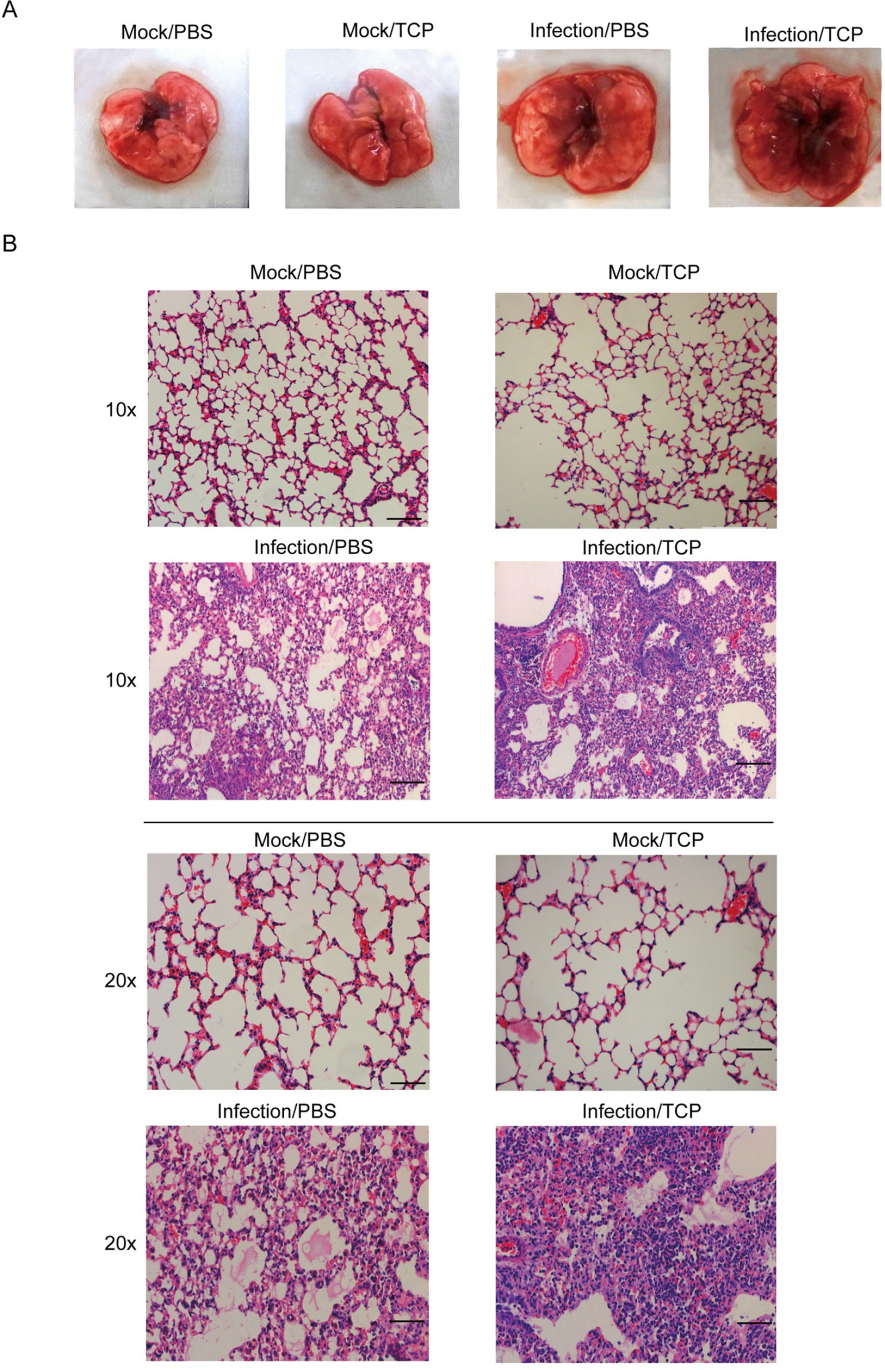
TCP treatment results in more severe lung injuries in IAV-infected mice. The PBS or TCP were admitted to Mock-infected or WSN-infected mice as above, and mice were sacrificed at Day 9 post-infection. Lung tissues from each group were collected and a representative result were shown in A. (B) The lungs were further sectioned, and stained with hematoxylin and eosin (H&E) for the histopathological analysis. Representative results were shown in B. Scale bar was 100μm in 10-time and 20-time enlargement.

To access the histological data, mice were mildly infected with WSN virus (500 pfu) wherein all the groups of animals could survive till at least day 9 post-infection. Mice were admitted with 10mg/kg TCP to magnify the effect of TCP. The lungs of the animals were isolated from each group for further analysis. As shown in Fig 5A, the structures of lung in both PBS- and TCP-treated mock-infected mice kept intact, while, the lung in TCP-treated WSN-infected mice had more bleeding and swelling as compared to PBS-treated WSN-infected mice. Histological data in Fig 5B indicated that TCP treatment in mock-infected mice did not induce observable toxicity. On the contrary, TCP treatment in WSN-infected mice induced marked lung pathology with massive infiltrating cells and hemorrhage in lung.

The data in all indicated that TCP treatment accelerated the disease course leading to more severe lethality and lung damage.

### TCP-induced disease aggravation is associated with IFITM3 activity and levels of IFITM3-K88me1

**Fig 6:**
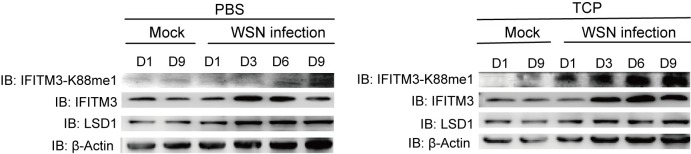
TCP treatment induces increased levels of IFITM3-K88me1 in IAV-infected mice. Mice (n = 3) in the indicated group were sacrificed at Day 1, Day 3, Day 6 and Day 9 respectively. Lung tissues were collected and homogenized for western blots to detect the levels of indicated proteins. Representative results are shown as above, data were repeated at least three times.

The lung tissues from sublethal infected mice were further collected to detect the level of IFITM3-K88me1 through Western blot. As compared to PBS-treated groups, the levels of IFITM3-K88me1 were significantly higher under TCP treatment, and IFITM3-K88me1 gradually increased during the course of the disease from Day 1 to Day 9 (Fig 6). Moreover, to establish the relationship of TCP and IFITM3, we used IFITM-knockout mice infected with sublethal infection of SC09 pandemic virus. The results showed that IFITM-knockout mice are more susceptible to infection and dead under low infection dose of virus (S4 Fig). The TCP treatment in these mice did not show further aggravation of disease neither in body-weight lost nor in lethality as compared to PBS treatment (S4 Fig). As IFITM3 makes a substantial contribution than any other IFITMs in IfitmDel knockout mice to IAV resistance *in vivo*[14], these data suggested that TCP-induced disease aggravation was associated with IFITM3 activity and high levels of IFITM3-K88me1.

### The disassociation of LSD1 from IFITM3 by IAV and VSV infections can counteract the function of IFITM3

**Fig 7:**
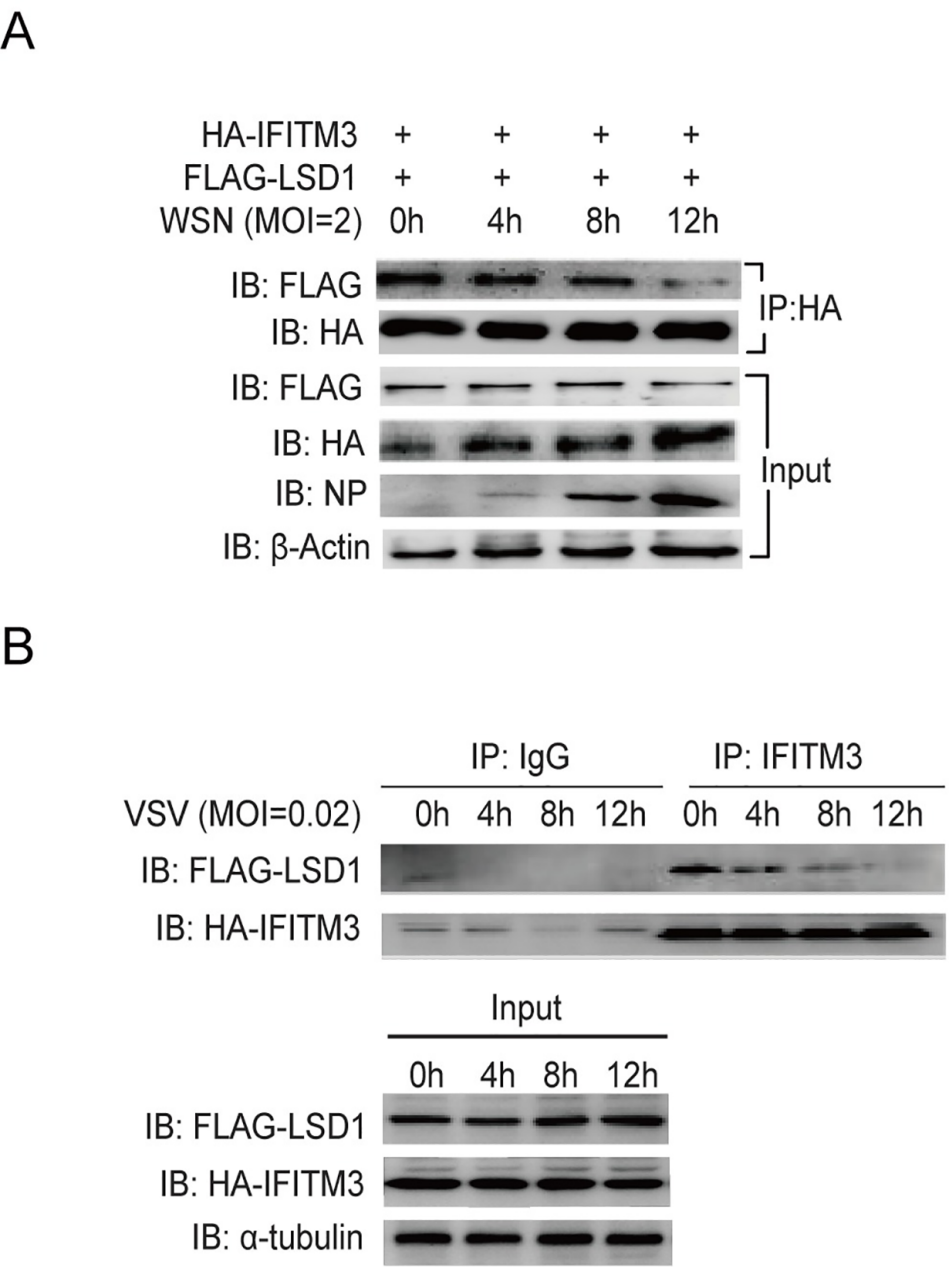
The disassociation of LSD1 from IFITM3 by IAV and VSV infections can counteract IFITM3. (A)Virus infection reduces the interaction between LSD1 and IFITM3. HEK293T cells were transfected with HA-IFITM3 and FLAG-LSD1. Forty-eight hours later, cells were infected with WSN virus at MOI = 2. Cells were then collected at the indicated time-point post-infection and were immunoprecipitated with anti-HA antibodies plus protein A/G beads. HA-IFITM3 and FLAG-LSD1 were detected as above, and levels of viral NP protein and β-Actin were shown in whole cell lysates (Input). (B) HEK293T cells grown in six-well plates were transfected with 0.5μg HA-IFITM3 and 1μg FLAG-LSD1. Forty-eight hours later, cells were infected with VSV at MOI = 0.02. Cells were then collected at the indicated time-point post-infection and were immunoprecipitated with 1μg anti-IFITM3 antibody plus protein A/G beads. Protein blots were probed with the antibodies as indicated, data were repeated at least three times.

As we observed that the IFITM3-K88me1 levels increased gradually in WSN-infected mice even without TCP treatment, we suspected that the virus may trigger IFITM3 methylation by disassociating LSD1 from IFITM3. To test this, HEK293T cells were transfected with HA-IFITM3 and FLAG-LSD1, and then infected with WSN virus. The data in Fig 7A showed that WSN infection heavily disassociated the interaction between IFITM3 and LSD1 in a time-dependent manner. This phenomenon was also observed in VSV-infected cells (Fig 7B). At 12h post-VSV-infection, the interaction between LSD1 and IFITIM3 was reduced compared with 0h post-infection (Fig 7B). Together with our previous findings that IAV or VSV infection increased the levels of IFITM3-K88me1 gradually upon infection[16], the data in all suggested that virus may develop strategies to comprise the action of type I IFN by inhibiting the binding of LSD1 to IFITM3.

**Fig 8:**
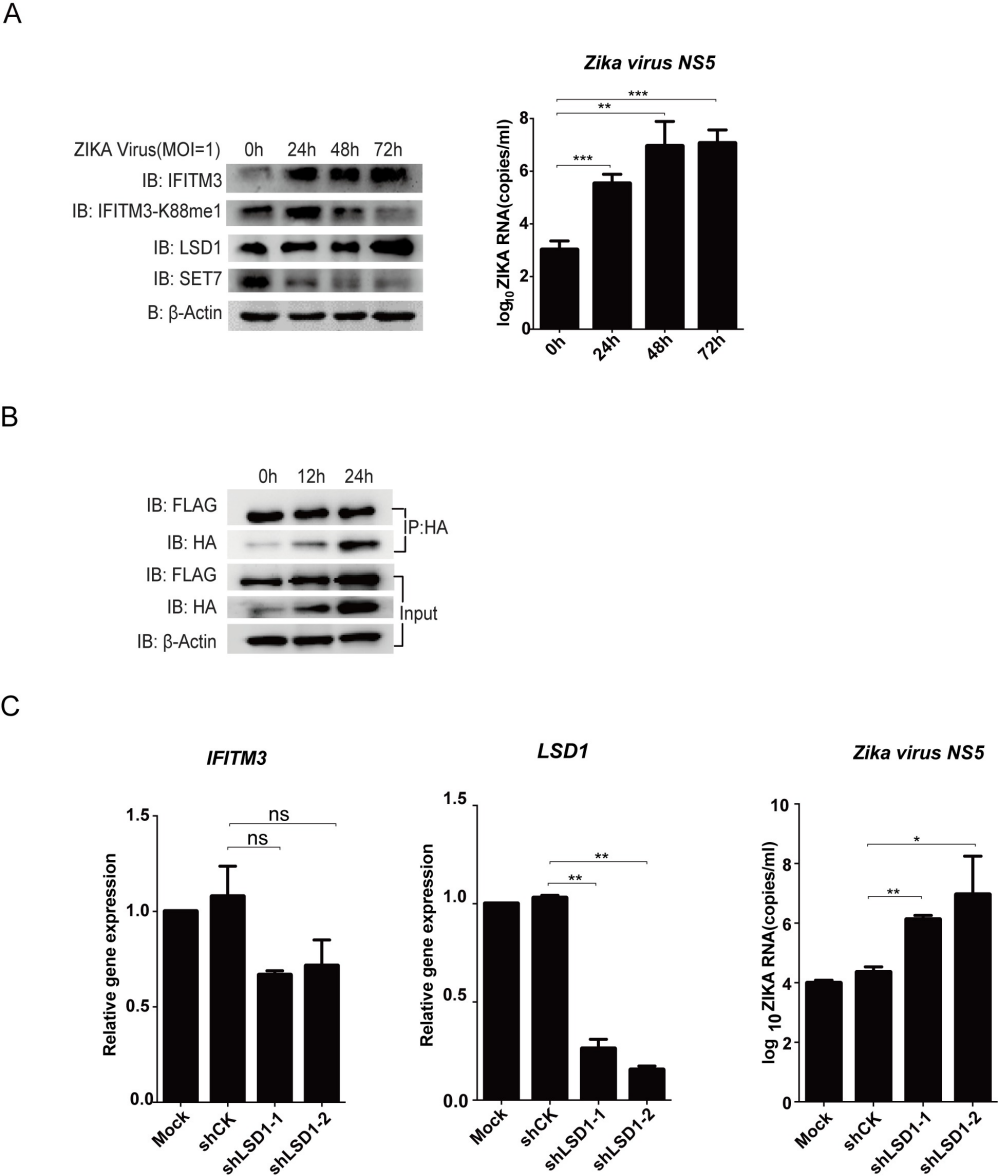
Zika virus infection triggers demethylation of IFITM3. (A) Zika virus infection increases the monomethylation of IFITM3. Human neural progenitor cells (hNPC) grown in six-well plates were infected with Zika virus at MOI = 1. Cells were then collected at the indicated time-point post-infection for western blot to detect the levels of IFITM3 and IFITM3-K88me1 or for qPCR to detect the *NS5* levels of Zika virus. The levels of IFITM3, IFITM3-K88me1, LSD1, SET7 and β-Actin were shown in whole cell lysates. Representative results are shown as above, data were repeated at least three times. The virus growth pattern is shown by Zika virus RNA copies on the right. (B) The levels of IFITM3-associated LSD1 keeps stable under Zika virus infection. Huh7 cells were transfected with HA-IFITM3 and FLAG-LSD1. Forty-eight hours later, cells were infected with Zika virus at MOI = 1. Cells were then collected at the indicated time-point post-infection and were immunoprecipitated with anti-HA antibodies plus protein A/G beads. HA-IFITM3 and FLAG-LSD1 were detected as above, and levels of β-Actin were shown in whole cell lysates (Input). Data were repeated at least three times. (C) Knockdown of LSD1 promotes multiplication of Zika virus. Lentivirus-packaged shRNAs against LSD1 (sh*LSD1*) were transduced into Huh7 cells. None-transduced Huh7 cells are included as control (Mock). The media was changed to fresh DMEM media 24h later. After another 24h, part of the cells was collected to check the knockdown efficiency of shRNAs (left and middle panel), the same time, equal number of 1×10^6^ cells were transferred to six-well plates and then treated with IFNα (200U/ml) to induce the expression of IFITM3. Twenty-four hours after IFNα treatment. The cells were infected with Zika virus at MOI = 1 and were then collected at 72h post-infection for qPCR to detect the *NS5* levels of Zika virus (right pannel). All data are representative of more than three independent experiments, and are shown by the mean value with +s.d. ns, *p* >0.05; *, *p* <0.05; **, *p* <0.01; ***, *p* <0.001.

However, when we tested Zika virus, the recently re-emerged arbovirus associated with microcephaly, we found that the levels of IFITM3-K88me1 were reduced upon infection even though the expression levels of IFITM3 increased (Fig 8A). Correspondingly, increased LSD1 expression and decreased SET7 expression were detected under Zika infection (Fig 8A). In addition, IFITM3-associated LSD1 levels did not decrease as fast as in IAV and VSV infections indicating that Zika virus did not disrupt LSD1/IFITM3 interaction as efficiently as VSV and IAV (Fig 8B). Nevertheless, knocking down LSD1 by shRNAs enhanced virus replication indicating that LSD1 is still anti-viral active in Zika-infected cells (Fig 8C). The result suggested that Zika virus was not able to counteract IFITM3 antiviral activity though methylation, and therefore could be more sensitive to IFITM3 as compared to IAV or VSV.

## Discussion

*IFITM3* is an ISG gene that presents broad-reactive anti-RNA virus activities[24]. It has been known post-translational modifications of IFITM3 would regulate its action against virus infection[25]. However, little is known about the responsible host enzymes catalyzing these post-translational modifications on IFITM3 and how important these modifications are in host protection against virus infection. In the current study, we reveal that LSD1 is the lysine demethyltransferase of IFITM3 at K88, which positively regulates the anti-RNA virus activity of IFITM3. Namely, TCP, a LSD1 inhibitor triggers more severe disease outcome in sub-lethal challenged IAV-infected mice, resulting in severe body weight loss, lethality and lung injuries. These data indicate that LSD1 contributes to control a mild infection through activating IFITM3 by K88 demethylation.

Lysine 88 on IFITM3 is located in the CIL domain which is exposed to the cytoplasma, so that it is highly accessible for cellular lysine modification machinery. We identified here and previously that K88 was methylated endogenously, which negatively regulates IFITM3 anti-viral activity[16]. Methylation on the lysine residue would neutralize the positive charge of the lysine motif, and therefore may change the protein-protein interactions. It is proposed that the IFITM3 complex network formed by polymerization between IFITM3 molecules may reduce the membrane fluidity which limits virus fusion with the host cell membrane[26]. It is possible that methylation on CIL domain may interfere with IFITM3 complex to loosen the IFITM3 network, so that fusion between cellular membrane and viral membrane is more feasible. Methylation may also change the interaction between IFITM3 and other host proteins to inhibit its anti-viral activity.

**Fig 9:**
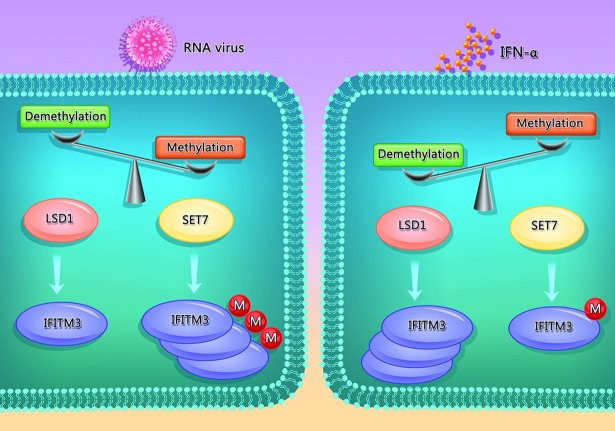
Reciprocal regulation of IFITM3 monomethylation by LSD1 and SET7. In the normal circumstances, there is a balance between the lysine methylation and demethylation of IFITM3 at K88. Under virus infection, the methylation of IFITM3 is increasing. On the contrary, type I IFN signaling will recruit LSD1 to demethylate K88me1 on IFITM3 to promote its antiviral activity during RNA virus infection. Our findings suggest that targeting the enzymatic activities of IFITM3 methylation modification enzymes can be useful to fight RNA virus infections.

Based on our previous data and current results, we suggest that LSD1 and SET7 is a pair of enzymatic modulators to regulate K88 methylation of IFITM3. The interaction between IFITM3 and LSD1 is found to be enhanced by IFNα, which in turn triggers K88 demethylation. As mRNA levels of LSD1 were stable under IFNα treatment (S5 Fig), the decrease of IFITM3-K88me1 is a response to IFNα instead of a simply consequence of increased abundance of LSD1. On the contrary, when cells are infected by RNA virus, such as VSV and IAV, the level of IFITM3-K88me1 was increased associated with less interaction between LSD1 and IFITM3. Therefore, IFNα signals strengthen host restriction factors through demethylating K88me1 on IFITM3 by LSD1, and virus infection counteract host restriction factors by promoting methylation of IFITM3 at K88. A balance between the lysine methylation and demethylation of IFITM3 at K88 may exist to maintain the cell homeostasis, and this balance may be broken under virus infection. A schematic working model was proposed and shown in Fig 9.

Zika virus has emerged as a severe health threat with a rapidly expanding range. IFITM3 is reported to be able to inhibit Zika virus infection in the early viral replication cycle[27]. In our study, Zika virus seems more susceptible to IFITM3-mediated host restriction because it cannot neutralize the anti-viral activity of IFITM3 through methylation. It is possible that further adaptation is required for Zika virus to counteract IFITM3, and viruses like IAV and VSV which circulate for a long history in humans had already well adapted to develop anti-IFITM3 strategies to methylate IFITM3.

To date, several studies have shown that post-translational modifications on IFITM3 affect its anti-viral activity in infected cells[25]. However, no evidence has been previously shown to demonstrate the important role of these modifications in infected animals *in vivo*. Our previous study has unexpectedly shown that IFITM3 may not limit but rather promote the progression of DNA virus such as HCMV by facilitating the formation of the virion assembly compartment during infection in cell culture[28]. Other studies have also shown *in vivo* that LSD1 inhibitor could be protective for host against DNA virus such as HSV infection[3, 4]. In this study, we have found that by opposition to its effect in anti-DNA virus infection, the enzymatic activity of LSD1 is critical to restrict RNA virus infection by controlling the limited levels of IFITM3 K88me1 in infected tissues. We treated WSN-infected mice with a LSD1 inhibitor (TCP) to investigate the role of IFITM3-K88me1 in host anti-viral response, and found that TCP treatment resulted in aggravated pathology as observed by severe body weight loss and increased mortality under IAV infection. The level of methylation of IFITM3 at K88 in the lung tissues of TCP-treated mice was also much higher than that in PBS-treated infected mice. Our results suggest that methylation of IFITM3 plays an essential role in disease development in IAV-infected animals. To regulate methylation, such as to stimulate the expression and upregulation of LSD1, may provide a new strategy for anti-flu therapeutics. On the other hand, as TCP strongly inhibits IFITM3-mediated antiviral activity by targeting LSD1, it could be potentially applied as an adjuvant to increase RNA virus propagation in IFITM3-competent system so as to assist vaccine industry or infection models.

## Materials and methods

### Ethics statement

The animal experiments were approved by the Institutional Animal Care and Use Committee of the Institut Pasteur of Shanghai, Chinese Academy of Sciences (Animal protocol #A2015006). All animal care and use protocols were performed in accordance with the Regulations for the Administration of Affairs Concerning Experimental Animals approved by the State Council of People’s Republic of China.

### Animals

Female 6- to 8-week-old BALB/c mice were purchased from model animal center of Shanghai Life Science. IFITM^-/-^ mice were purchased from EMMA (*IfitmDel*) wherein the entire *Ifitm*1, 2, 3, 5, and 6 locus were deleted[29].

### Cell lines, plasmids, antibodies and reagents

Human neural precursor cells (hNPC)[30] was cultured in N2B27+bFGF medium composed of DMEM/F12 and Neuralbasal in a ratio of 1:1 supplemented with 0.5% N2 (Gibco), 1% B27 (Gibco), 1%NEAA (Gibco), 1% L-Glu (Gibco), and 20ng/ml bFGF (Gibco). Other cell lines used in these studies were cultured in Dulbecco’s modified Eagle’s medium (DMEM) (HyClone) supplemented with 10% fetal bovine serum (FBS) (ExCell Bology), 1% penicillin/streptomycin (Gibco). HEK293T cells (American Type Culture Collection) were transfected with appropriate plasmids using polyethylenimine (PEI) reagent (Polysciences) according to the manufacturer’s instructions. The plasmids del8.9 and VSV-G for lentivirus production were gifts from Prof. Ke Lan (Institut Pasteur of Shanghai). The full sequences of *IFITM3*, *SET7* and *LSD1* were amplified by PCR with cDNA from Hela cells (American Type Culture Collection) or HEK293T cells. These fragments were then cloned into HA-tagged, FLAG-tagged or non-tagged (pcDNA3.1) vectors by standard procedures. The IFITM3-K88R and -K88A mutants were constructed with the Quickchange II site-directed mutagenesis kit (Stratagene) according to the manufacturer’s standard procedures. sh*IFITM3-1*, sh*LSD1-1*, sh*LSD1-2*, sh*SET7-1*, sh*SET7-2* and sh*CK* were constructed in pLKO.1 using the following targeting sequence:

*IFITM3-1* (CCTGTTCAACACCCTCTTCAT);
*LSD1-1* (GCTCCAATACTGTTGGCACTA);
*LSD1-2* (CCAACAATTAGAAGCACCTTA);
*SET7-1* (GCAAACTGGCTACCCTTATGT);
*SET7-2* (GGGAGTTTACACTTACGAAGA);
*CK* (CAACAAGATGAAGAGCACCAA);

The antibodies used in this study were as follows: anti-HA (F-7, Santa Cruz Biotechnology), anti-FLAG (M2, Sigma-Aldrich), anti-LSD1 (Cell Signaling), anti-IFITM3 (11714-1-AP, Protein Tech Group, Inc.), anti-β-Actin (C1213, Sungene Biotech), anti-α-tubulin (DM1A, Sigma), anti-Influenza A NP (9G8, Sc-101352, Santa Cruz Biotechnology). Alexa Fluor anti-mouse 555 (Invitrogen-Molecular Probes), anti-rabbit 488 (Invitrogen-Molecular Probes) and anti-rat 633 (Invitrogen-Molecular Probes). The αK88me1 rabbit polyclonal antibody was generated by Abmart, raised toward the polypeptide SRDR (Kme1) MVGD. Protein A/G PLUS Agarose beads (A10001) were purchased from Santa Cruz Biotechnology. IFNα (recombinant human interferon α-2b) was purchased from Shanghai Hua Xin High Biotechnology Inc. The LSD1 inhibitor (TCP) was purchased from Sigma.

### Quantitative real-time PCR

Cells were collected and lysed in TRIzol Reagent (Invitrogen) and RNA was isolated according to the manufacturer’s instructions. Reverse transcription was performed with the PrimeScript RT regent kit (TaKaRa). The cDNA samples were used at 10ng/well in a 384-well plate and run in triplicate. PCR reactions were set up in 10μl volumes with SYBR Premix Ex Taq regent (TaKaRa) on an ABI Prism 7500 Sequence Detection System. GAPDH was used as the reference control for the target genes. The primers were listed below:

*IFITM3* (F: 5’-TCCCACGTACTCCAACTTCCA-3’, R: 5’-AGCACCAGAAACACGTGCACT-3’);
*LSD1* (F: 5’-CGGGCGAAGGTAGAGTACAG-3’, R: 5’-CGTCTCCATACCCTCCAGAA-3’);
*SET7* (F: 5’-AGTTCTCCAGGGCACGTATG-3’, R: 5’-TCTCCAGTCATCTCCCCATC-3’);
*GAPDH* (F: 5’-GAGTCAACGGATTTGGTCGT3’, R: 5’-GACAAGCTTCCCGTTCTCAG-3’);
*VSV-L* (F: 5’-TGATACAGTACAATTATTTTGGGAC-3’, R: 5’-GAGACTTTCTGTTACGGGATCTGG-3’);
*WSN* seg5_432[31] (F: 5’-AACGGCTGGTCTGACTCACATGAT-3’, R: 5’-AGTGAGCACATCCTGGGATCCATT-3’).
*Zika virus NS5* (F: 5’-CTCCAGGATGCAAGTCTAAG-3’, R:
5’-ACCCAGCAGGAACTTCAGGA-3’).

The relative gene expression is calculated by $2^{-\Delta \Delta C_{T}}$, where ΔΔ*C*_*T*_ = (*C*_*T,Target*_ − *C*_*T,Actin*_)_EG_– (*C*_*T,Target*_ − *C*_*T,Actin*_)_CG_, EG represents experimental group and CG represents control group.

### Lentivirus packaging and transduction

The shRNA sequences were synthesized by Shanghai Sunny Biotechnology Co.Ltd. Lentivirus package plasmids delta8.9 and VSV-G were co-transfected into HEK293T cells. Forty-eight-hour post-transfection, viral supernatants were harvested after centrifugation and filtration for downstream use.

### Co-immunoprecipitation (Co-IP) and immunoblotting

Cells were harvested and washed with ice-cold PBS and lysed in radio immunoprecipitation assay (RIPA) buffer (50 mM Tris/HCl [pH 7.4], 150mM NaCl, 1% Triton X-100, 0.5% sodium deoxycholate, 0.1% SDS, 1mM EDTA, 10% glycerol with 1 mM PMSF, 1 mM Na_3_VO_4_, 1 mM NaF and protease inhibitor [1:100, P8340, Sigma-Aldrich]) for 30min on ice. The lysates were incubated with antibody or affinity beads as indicated overnight at 4°C. The immunoprecipitations were separated by SDS-PAGE and analyzed by immunoblotting.

### Fluorescence microscopy

Hela cells grown on glass cover-slips were fixed in 2% paraformaldehyde (in phosphate-buffered saline) for 20min at room temperature and permeabilized with 0.1% Triton X-100 (in phosphate-buffered saline) for 15min at room temperature. Subsequently, the cells were blocked with 5% FBS (in phosphate-buffered saline, PBS) for 20min and incubated with the primary antibodies indicated below for 60min at room temperature. Primary antibodies to the following (and their dilutions) were used: anti-Myc (Mouse, 1:500), anti-HA (Rabbit, 1:500), and anti-FLAG (Rat, 1:1000). Cells were then washed with PBS and stained with Alexa Fluor-labeled anti-mouse, anti-rabbit and anti-rat secondary antibodies (1:1000;) for 20min at room temperature in dark and washed five times with 0.1% Triton X-100 in PBS after each antibody treatment. To visualize the nuclei, cells were counterstained with DAPI (4′, 6-diamidino-2-phenylindole; 1:5000; Beyotime) for 10min. Finally, labeled cells were mounted on slides with Mowio reagent (4–88, Chem. Bochem) overnight. Images were captured using a Leica TCS SP5 confocal laser scanning microscope.

### MBP pull-down assay

MBP, MBP-LSD1, and GST-IFITM3 were expressed in Rosetta/pLysS and purified by amylose resin or glutathione-Sepharose 4B (GE Healthcare). After dialysis, proteins were quantified by SDS/PAGE using BSA as the standard. MBP, GST-IFITM3, MBP-LSD1, and GST-IFITM3 were incubated for 2h at 4°C in buffer A [20mM Tris·HCl (pH 7.5), 200 mM NaCl, 5 mM β-mercaptoethanol, and 1 mM PMSF] and were then incubated with amylose resins for another 2h. Amylose resins were then washed extensively with buffer A, followed by SDS/PAGE.

### VSV virus production and infection

VSV (Indiana serotype, American Type Culture Collection). Recombinant VSV expressing green fluorescent protein (VSV-GFP)[32] was propagated in Vero cells after infection at a MOI = 0.01. Forty-eight hours later, supernatant was collected after centrifugation and then filtered through a 0.45μm filter (Millipore) and stored at -80°C. Virus infections were performed in the absence of serum for 1h, and then replaced with fully supplemented growth medium. Endpoint dilution assay was used to quantify VSV titers with log dilutions on 100% confluent Vero cells plated in 96-well plates.

### A/WSN/33 (H1N1) virus production and infection

Recombinant viruses were generated by plasmid-based reverse genetics followed by two-round plaque purification and propagation in MDCK cells (American Type Culture Collection) as described previously[33]. Briefly, eight viral cDNAs (pHW2000-WSN-NP, pHW2000-WSN-PB1, pHW2000-WSN-PB2, pHW2000-WSN-pA, pHW2000-WSN-NS, pHW2000-WSN-M, pHW2000-WSN-HA, pHW2000-WSN-NA) from A/WSN/33 (H1N1) were gifts from Hans-Dieter Klenk (University of Marburg), which were co-transfected into HEK293T cells. Supernatant was then transferred onto MDCK cells 72h post-transfection. Virus multiplied from MDCK cells was further purified by a two-round plaque assay followed by multiplication. To examine the broad-reactive activity of IFITM3 against virus infections, A549 cells (American Type Culture Collection) were transfected with shRNAs followed by virus infection at MOI = 5 for 30min in PBS 0.2% BSA. After 3×washes with PBS, infected cells were then cultured in complete DMEM culture media supplemented with 2% FBS. Cell extracts were collected 8h post-infection and analyzed by Western blot and qPCR.

### Zika virus production and infection

Zika virus SZ-WIV001 (KU963796) was kindly provided by Wuhan institute Virology CAS[34]. Zika virus was amplified in VeroE6 cells (American Type Culture Collection). Cells were infected with Zika virus in the absence of FBS for 1h. Media were then removed, and substituted by the appropriate culture medium, supplemented with 3% FBS and 20mM HEPES. Infected cell cultures were observed daily for detection of cytopathic effects (CPE, day 3–4 post-infection). Supernatants were collected when CPE occurs, stored at -80°C after centrifugation for virus stock. Human neural precursor cells (hNPC) were infected with Zika virus at MOI = 1, cell extracts are collected 24h, 48h and 72h post-infection and analyzed by Western Blot. Huh7 cells were first transfected with IFITM3 and LSD1 or transducted with lentivirus derived shRNAs and then infected with Zika virus at MOI = 1. Cell supernatants were further collected to titrate virus RNA copies by real-time PCR.

### Mice infection experiment

Female 6- to 8-week-old BALB/c mice were randomly divided into 4 groups: PBS-mock group, TCP-mock group, PBS-WSN treated group, and TCP-WSN treated group.

A/WSN/33 of 10000 pfu (lethal dose) was given in 50μl PBS per mouse intranasal. The body weights of mice were monitored throughout the infection time course. After sublethal infection (500 pfu), mice would be sacrificed at Day 1, Day 3, Day 6, and Day 9 respectively to collect lung tissues for the detection of IFITM3-K88me1 through Western blotting method and of residue virus titers through plaque titration on MDCK cells. At Day 9, the lung tissues were further fixed in neutral formalin, embedded in paraffin, sectioned, and stained with hematoxylin and eosin (H&E).

A/Sichuan/1/2009 (H1N1) (SC09) virus (kindly provided by Prof. Yuelong Shu in China CDC) were also used to infect the mice at 300 pfu, and the body weights of mice were monitored throughout the infection time course.

### Statistical analysis

Data were analyzed by Student’s *t* test. A *P* value of < 0.05 was considered to be statistically significant difference. ns, *p* >0.05; *, *p* <0.05; **, *p* <0.01; ***, *p* <0.001. Statistical analyses were done using GraphPad Prism. Error bars represent standard deviation (s.d.).

## Supporting information

S1 Fig Co-localization of IFITM3 and LSD1. Hela cells plated on cover glasses were transfected with FLAG-LSD1, Myc-IFITM3 or HA-IFITM3-K88R/A mutants. Twenty-four hours later, the cells were fixed and processed to immunofluorescence staining with mouse anti-Myc, rabbit anti-HA and rat anti-FLAG as the first antibodies respectively. Alexa fluor anti-mouse 555, anti-rabbit 488 and anti-rat 633 were used as the secondary antibodies accordingly. The nucleuses were stained with DAPI. Representative results are shown.
(TIF)

S2 Fig The double knockdown efficacy of IFITM3 and LSD1/SET7 under VSV infection. Experimental procedure is shown in A. Lentivirus-packaged shRNAs against either SET7 (sh*SET7*) and IFITM3 (sh*IFITM3*) or LSD1 (sh*LSD1*) and IFITM3 (sh*IFITM3*) were transduced into HEK293T cells. None-transduced 293T cells are included as control (Mock).The media was changed to fresh DMEM media 24h later. After another 24h, equal number of 5×10^5^ cells were transferred to twelve-well plates and then treated with IFNα (100U/ml) to induce the expression of IFITM3. Mock cells were treated without IFNα. Twenty-four hours after IFNα treatment, the cells were infected with VSV at MOI = 0.02 and were then collected at 12h post-infection for real-time PCR and western blotting. (B) The qPCR analyses of the mRNA levels of IFITM3, SET7 or LSD1 respectively. (C) The western blots of IFITM3 and IFITM3-K88me1. All data are representative of more than three independent experiments, and are shown by the mean value with+s.d. ns, *p* >0.05; *, *p* <0.05; **, *p* <0.01; ***, *p* <0.001.
(TIF)

S3 Fig The effects of double knockdown of IFITM3 and LSD1/SET7 under IAV infection. Experimental procedure is shown in A. Lentivirus-packaged shRNAs against either SET7 (sh*SET7*) and IFITM3 (sh*IFITM3*) or LSD1 (sh*LSD1*) and IFITM3 (sh*IFITM3*) were transduced into A549 cells. None-transduced A549 cells are included as control (Mock). The media was changed to fresh DMEM media 24h later. After another 24h, equal number of 5×10^5^ cells were transferred to twelve-well plates and then treated with IFNα (200U/ml) to induce the expression of IFITM3. Mock cells were treated without IFNα. Twenty-four hours after IFNα treatment, the cells from all the groups were infected with WSN at MOI = 5 and were then collected at 8h post-infection for real-time PCR and western blotting. (B) The qPCR analyses of the mRNA levels of IFITM3, SET7 or LSD1 respectively. (C) The western blots of IFITM3 and IFITM3-K88me1. All data are representative of more than three independent experiments, and are shown by the mean value with +s.d. ns, *p* >0.05; *, *p* <0.05; **, *p* <0.01; ***, *p* <0.001.
(TIF)

S4 Fig TCP treatment has limited effect in IFITM-knockout mice under IAV-infection. The mice (n = 3) of wide type (WT) and IFITM^-/-^ (KO) were pretreated with PBS (100μl/kg) or TCP (5mg/kg) through intraperitoneally injection on day 0 (D0). One hour later, mice were infected with 300 pfu of A/Sichuan/1/2009 (H1N1) in 50μl PBS or 50μl PBS (mock) intranasally. All mice were injected intraperitoneally with PBS (100μl/kg) or TCP (5mg/kg) once a day. (A) The lung tissues were homogenized and subjected to western blots for IFITM3 expression. (B) The body weights of mice were monitored throughout the infection time course from Day 0 to Day 14. The survival curve of mice was shown in C. For WT mice, the body-weight differences between PBS-infection and TCP-infection groups were significant (p<0.05) from Day 2 to Day 7. However, for IFITM^-/-^ mice, there were no significant differences in body weights between PBS-infection and TCP-infection groups.
(TIF)

S5 Fig The mRNA levels of LSD1 are stable post-IFNα treatment. HEK293T cells grown in six-well plates were treated with IFNα (200U/ml) for the indicated time periods and were then collected following by qPCR analyses of the mRNA levels of LSD1. The data are shown as mean + s.d. of three independent experiments. ns, p >0.05.
(TIF)
