# Correction: Histone demethylase LSD1 restricts influenza A virus infection by erasing IFITM3-K88 monomethylation

**DOI:** 10.1371/journal.ppat.1007037

**Published:** 2018-04-30

**Authors:** Jiaoyu Shan, Binbin Zhao, Zhao Shan, Jia Nie, Rong Deng, Rui Xiong, Andy Tsun, Weiqi Pan, Hanzhi Zhao, Ling Chen, Ying Jin, Zhikang Qian, Kawing Lui, Rui Liang, Dan Li, Bing Sun, Dimitri Lavillette, Ke Xu, Bin Li

There is an error in affiliation 1 for authors Jiaoyu Shan, Binbin Zhao, Zhao Shan, Jia Nie, Rong Deng, Andy Tsun, Kawing Lui, Rui Liang. Affiliation 1 should be:

CAS Center for Excellence in Molecular Cell Science, CAS Key Laboratory of Molecular Virology and Immunology, Unit of Molecular Immunology, Institut Pasteur of Shanghai, Shanghai Institutes for Biological Sciences, Chinese Academy of Sciences; University of Chinese Academy of Sciences, Shanghai, China

## References

[1] Shan J, Zhao B, Shan Z, Nie J, Deng R, Xiong R, et al (2017) Histone demethylase LSD1 restricts influenza A virus infection by erasing IFITM3-K88 monomethylation. PLoS Pathog 13(12): e1006773 https://doi.org/10.1371/journal.ppat.1006773 29281729 10.1371/journal.ppat.1006773 PMC5760097

