# Correction: Histone demethylase LSD1 restricts influenza A virus infection by erasing IFITM3-K88 monomethylation

**DOI:** 10.1371/journal.ppat.1009359

**Published:** 2021-02-22

**Authors:** Jiaoyu Shan, Binbin Zhao, Zhao Shan, Jia Nie, Rong Deng, Rui Xiong, Andy Tsun, Weiqi Pan, Hanzhi Zhao, Ling Chen, Ying Jin, Zhikang Qian, Kawing Lui, Rui Liang, Dan Li, Bing Sun, Dimitri Lavillette, Ke Xu, Bin Li

There are errors in Figs 1D and 3C. The incorrect reference protein blot images were included for Fig 1D; IB: α-tubulin and Fig 3C; IB: α-tubulin. The reference protein blot in Fig 3C is also mis-labelled as IB: α-tubulin and should be IB: Histone. Please see the correct figures here:

**Fig 1:**
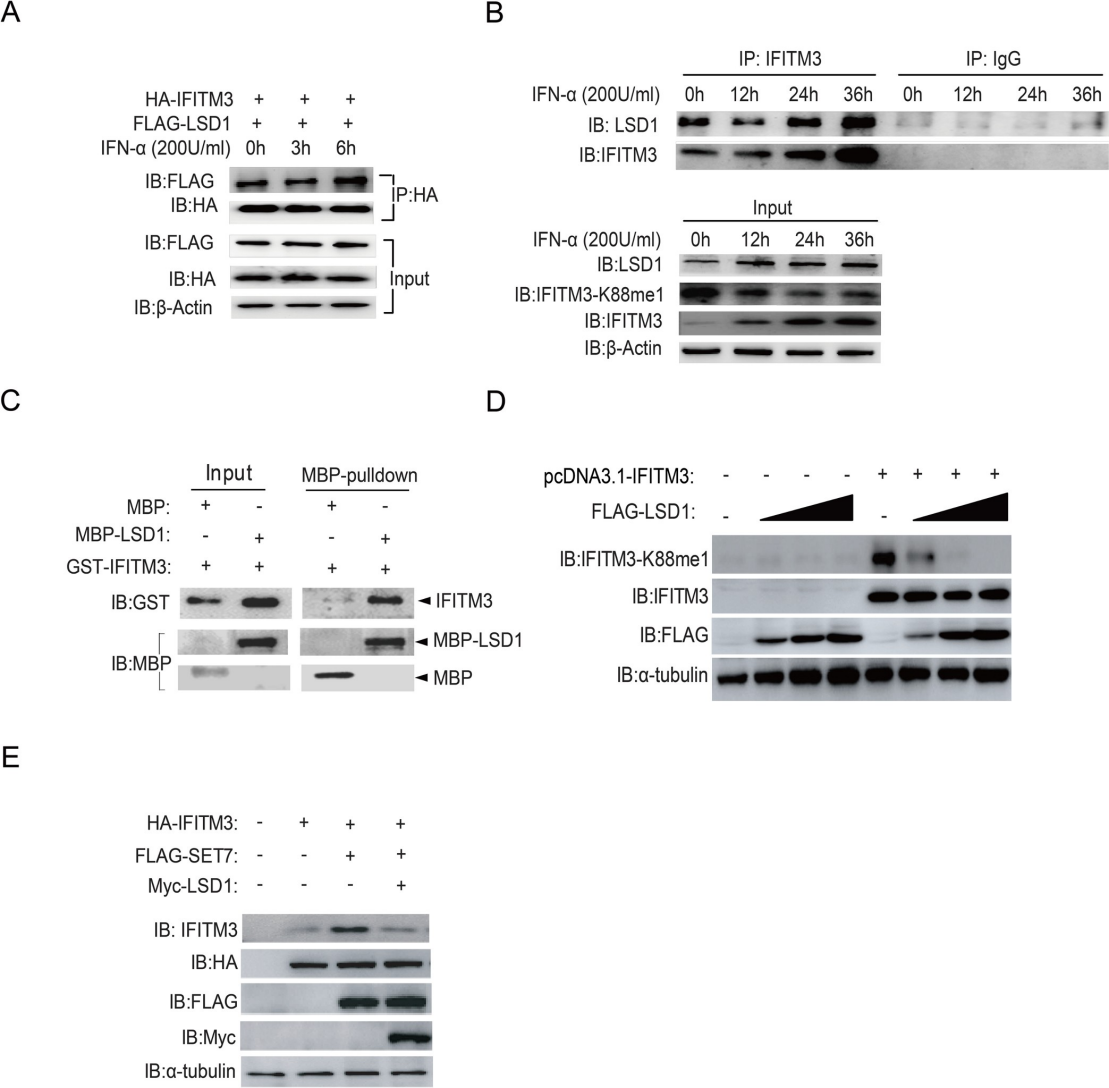
LSD1 catalyzes lysine demethylation of IFITM3 at K88. (A) HEK293T cells grown in six-well plates were transfected with HA-IFITM3 and FLAG-LSD1. Forty-eight hours later, cells were treated with IFNα (200U/ml) for the indicated time periods. IFITM3 was immunoprecipitation (IP) by anti-HA antibodies and LSD1 was probed with indicated antibody. The expression levels of HA-IFITM3, FLAG-LSD1 and β-Actin were shown as loading control (Input). (B) HEK293T cells grown in six-well plates were treated with IFNα (200U/ml) for the indicated time periods and were then collected to perform immunoprecipitation (IP) of endogenous IFITM3. The levels of LSD1 and IFITM3 were detected by immunoblotting with anti-LSD1 and anti-IFITM3 antibodies. Proteins levels in whole cell lysates were shown as Input. (C) The interaction of LSD1 and IFITM3 is direct. A pull down assay was performed with recombinant MBP-LSD1, MBP, and GST-IFITM3 as indicated. Immunoblotting (IB) was used to show protein levels. (D) LSD1 down-regulates monomethylation of IFITM3 at K88 in a dose dependent manner. HEK293T cells were transfected with pcDNA3.1-IFITM3 and increasing amounts of FLAG-LSD1 plasmids (0.6μg, 1.5μg or 2.4μg). Forty-eight hours later, cells were collected and levels of IFITM3-K88me1 were determined by western blotting. (E) The demethylase activity of LSD1 is antagonized with SET7. FLAG-tagged SET7, Myc-tagged LSD1 and HA-tagged IFITM3 were co-transfected into HEK293T cells as indicated. After 48h, the cells were collected and protein levels were detected by western blotting.

**Fig 3:**
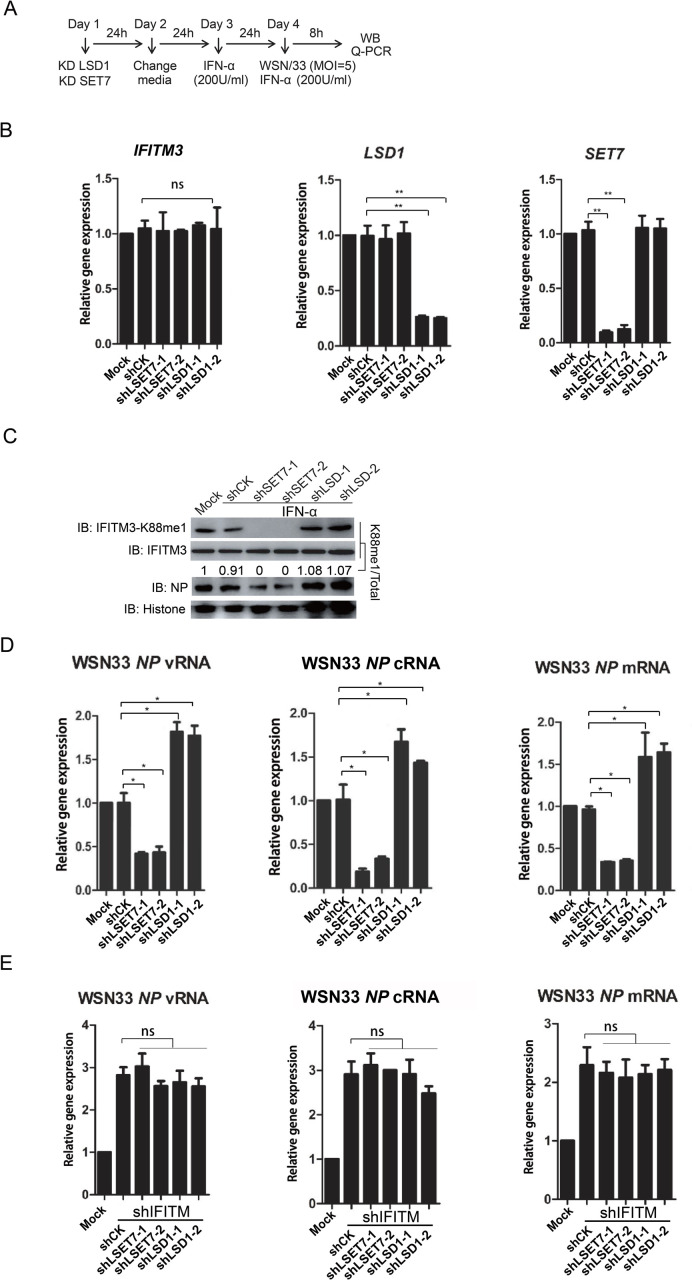
LSD1 enhances the antiviral activity of IFITM3 against IAV infection. Experimental procedure is shown in A. (B) Knockdown efficiency of shRNAs against SET7 and LSD1. HEK293T cells were infected by lentivirus coding shRNA against either SET7 (shSET7) or LSD1 (shLSD1) followed qPCR analyses of the mRNA levels of IFITM3, SET7 or LSD1 respectively. (C and D) Knockdown of LSD1 promotes the methylation of IFITM3 at K88, whereas, knockdown of SET7 reduces IFITM3-K88me1. Lentivirus-packaged shRNAs against either SET7 (shSET7) or LSD1 (shLSD1) were transduced into A549 cells. None-transduced A549 cells are included as control (Mock). The media was changed to fresh DMEM media 24h later. After another 24h, equal number of 5×105 cells were transferred to twelve-well plates and then treated with IFNα (200U/ml) to induce the expression of IFITM3. Mock cells were treated without IFNα. Twenty-four hours after IFNα treatment, the cells were infected with WSN at MOI = 5 and were then collected at 8h post-infection for western blotting to detect the levels of IFITM3 and IFITM3-K88me1 (C) or for qPCR to detect the vRNA, cRNA and mRNA levels of viral gene (D). (E) The effects of LSD1 and SET7 are dependent of IFITM3 expression. Experimental procedure is same as procedure shown in A except that lentivirus-packaged shRNAs against either SET7 (shSET7) and IFITM3 (shIFITM3) or LSD1 (shLSD1) and IFITM3 (shIFITM3) were transduced into A549 cells on the Day 1. Cells were then collected at 8h post-infection for qPCR to detect the vRNA, cRNA and mRNA levels of viral gene. All data are representative of more than three independent experiments, and are shown by the mean value with +s.d. ns, p >0.05; *, p <0.05; **, p <0.01; ***, p <0.001.
