# Supplementary figures and images for: Histone demethylase LSD1 restricts influenza A virus infection by erasing IFITM3-K88 monomethylation
Source: PLoS Pathog. 2017 Dec 27;13(12):e1006773. doi: 10.1371/journal.ppat.1006773 (PMC5760097; doi:10.1371/journal.ppat.1006773)

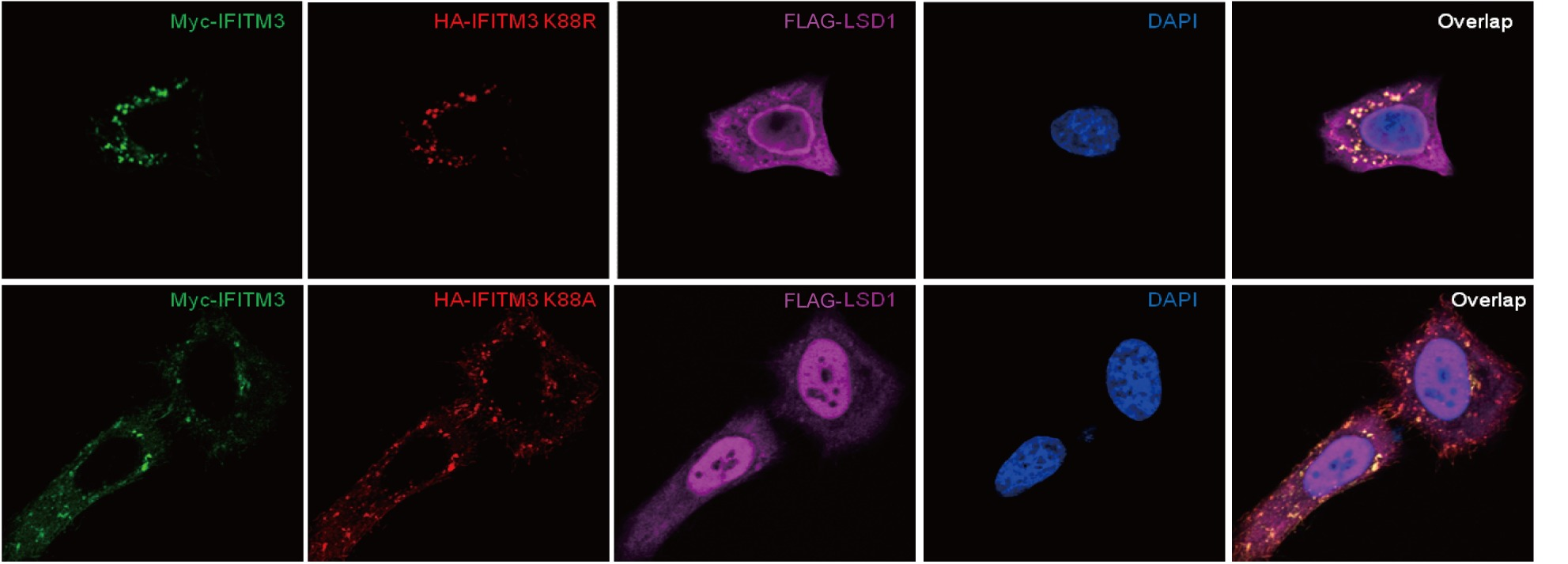

Supplement: S1 Fig — Hela cells plated on cover glasses were transfected with FLAG-LSD1, Myc-IFITM3 or HA-IFITM3-K88R/A mutants. Twenty-four hours later, the cells were fixed and processed to immunofluorescence staining with mouse anti-Myc, rabbit anti-HA and rat anti-FLAG as the first antibodies respectively. Alexa fluor anti-mouse 555, anti-rabbit 488 and anti-rat 633 were used as the secondary antibodies accordingly. The nucleuses were stained with DAPI. Representative results are shown. (TIF) [file ppat.1006773.s001.tif]

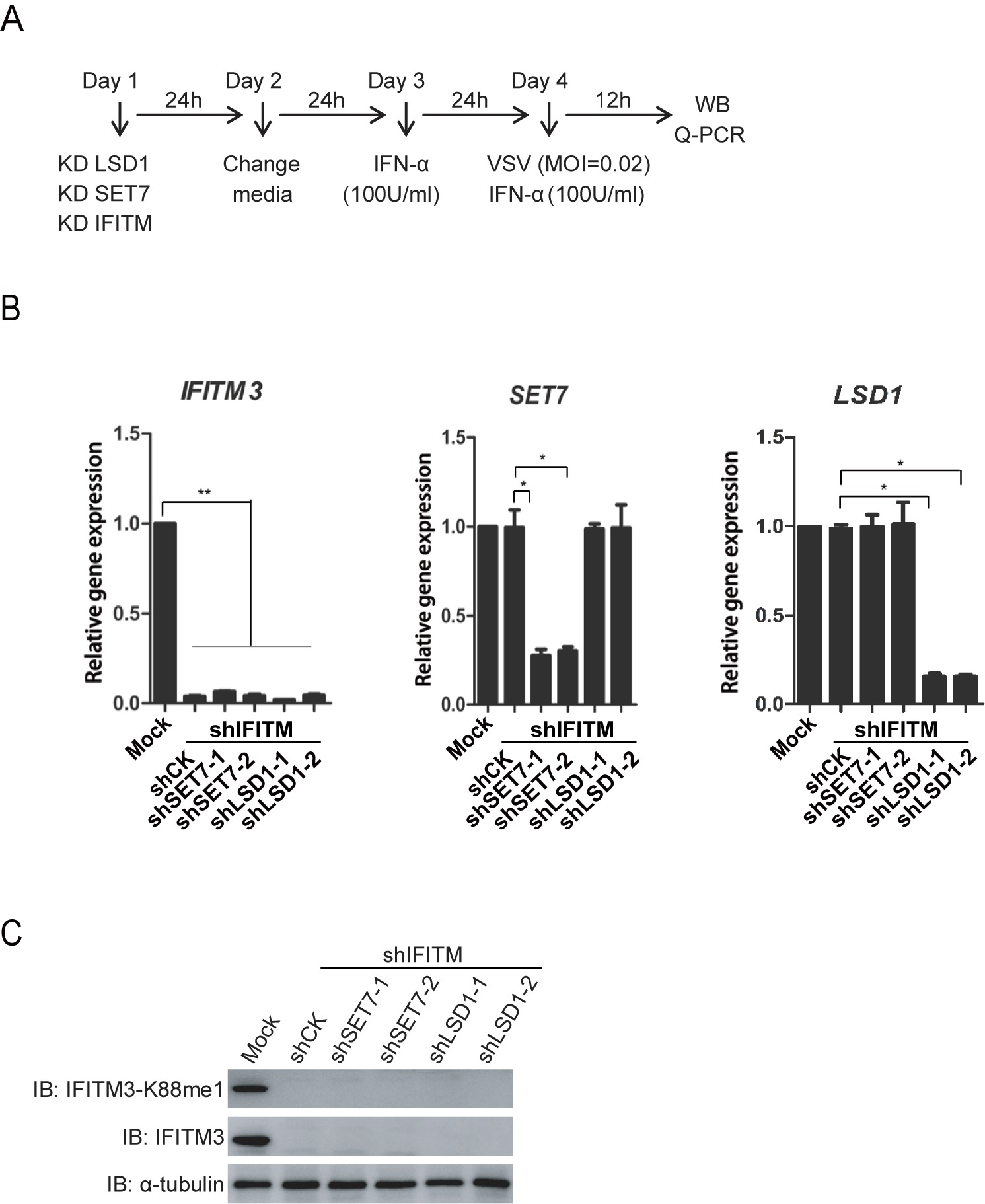

Supplement: S2 Fig — Experimental procedure is shown in A. Lentivirus-packaged shRNAs against either SET7 (shSET7) and IFITM3 (shIFITM3) or LSD1 (shLSD1) and IFITM3 (shIFITM3) were transduced into HEK293T cells. None-transduced 293T cells are included as control (Mock).The media was changed to fresh DMEM media 24h later. After another 24h, equal number of 5×10^5^ cells were transferred to twelve-well plates and then treated with IFNα (100U/ml) to induce the expression of IFITM3. Mock cells were treated without IFNα. Twenty-four hours after IFNα treatment, the cells were infected with VSV at MOI = 0.02 and were then collected at 12h post-infection for real-time PCR and western blotting. (B) The qPCR analyses of the mRNA levels of IFITM3, SET7 or LSD1 respectively. (C) The western blots of IFITM3 and IFITM3-K88me1. All data are representative of more than three independent experiments, and are shown by the mean value with+s.d. ns, p >0.05; *, p <0.05; **, p <0.01; ***, p <0.001. (TIF) [file ppat.1006773.s002.tif]

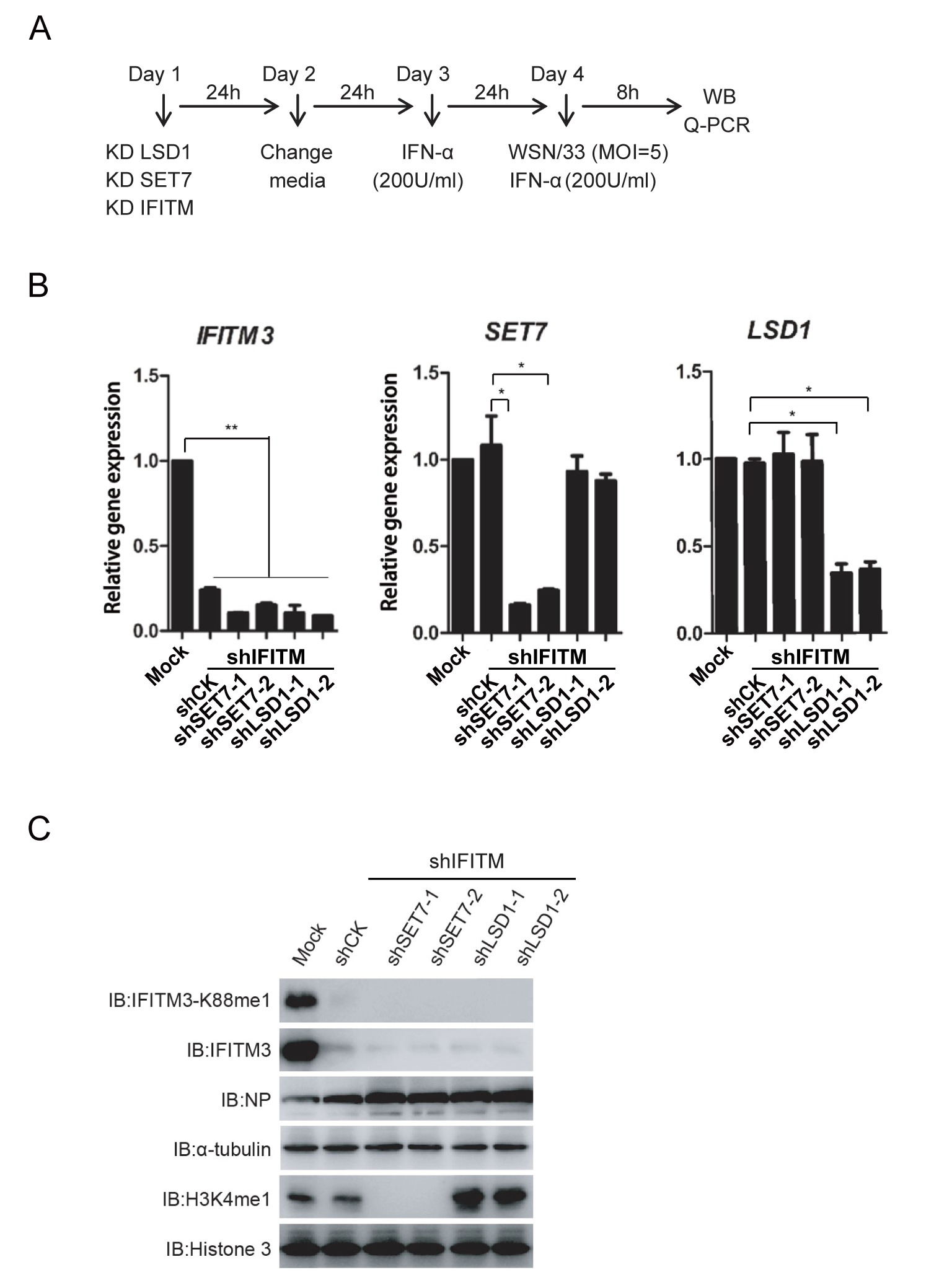

Supplement: S3 Fig — Experimental procedure is shown in A. Lentivirus-packaged shRNAs against either SET7 (shSET7) and IFITM3 (shIFITM3) or LSD1 (shLSD1) and IFITM3 (shIFITM3) were transduced into A549 cells. None-transduced A549 cells are included as control (Mock). The media was changed to fresh DMEM media 24h later. After another 24h, equal number of 5×10^5^ cells were transferred to twelve-well plates and then treated with IFNα (200U/ml) to induce the expression of IFITM3. Mock cells were treated without IFNα. Twenty-four hours after IFNα treatment, the cells from all the groups were infected with WSN at MOI = 5 and were then collected at 8h post-infection for real-time PCR and western blotting. (B) The qPCR analyses of the mRNA levels of IFITM3, SET7 or LSD1 respectively. (C) The western blots of IFITM3 and IFITM3-K88me1. All data are representative of more than three independent experiments, and are shown by the mean value with +s.d. ns, p >0.05; *, p <0.05; **, p <0.01; ***, p <0.001. (TIF) [file ppat.1006773.s003.tif]

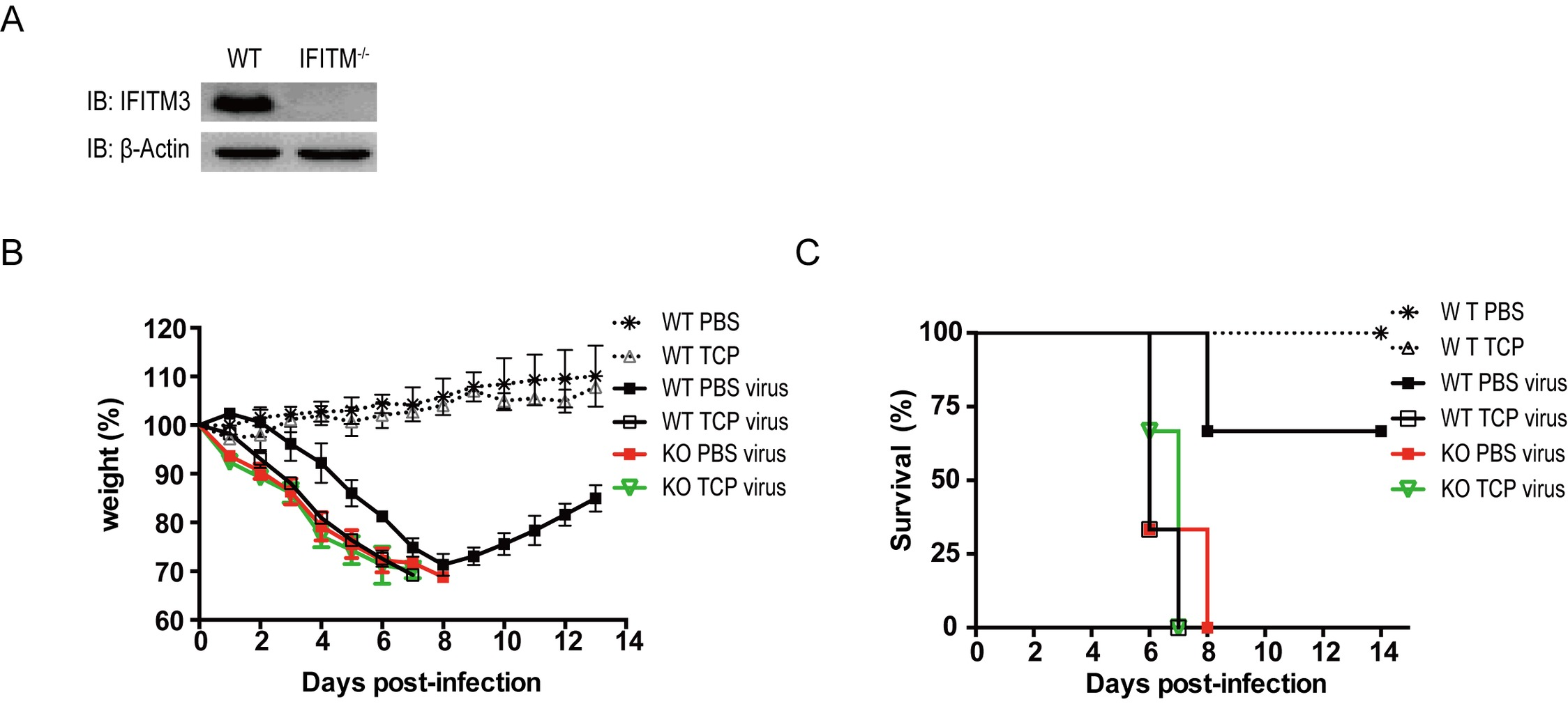

Supplement: S4 Fig — The mice (n = 3) of wide type (WT) and IFITM-/- (KO) were pretreated with PBS (100μl/kg) or TCP (5mg/kg) through intraperitoneally injection on day 0 (D0). One hour later, mice were infected with 300 pfu of A/Sichuan/1/2009 (H1N1) in 50μl PBS or 50μl PBS (mock) intranasally. All mice were injected intraperitoneally with PBS (100μl/kg) or TCP (5mg/kg) once a day. (A) The lung tissues were homogenized and subjected to western blots for IFITM3 expression. (B) The body weights of mice were monitored throughout the infection time course from Day 0 to Day 14. The survival curve of mice was shown in C. For WT mice, the body-weight differences between PBS-infection and TCP-infection groups were significant (p<0.05) from Day 2 to Day 7. However, for IFITM-/- mice, there were no significant differences in body weights between PBS-infection and TCP-infection groups. (TIF) [file ppat.1006773.s004.tif]

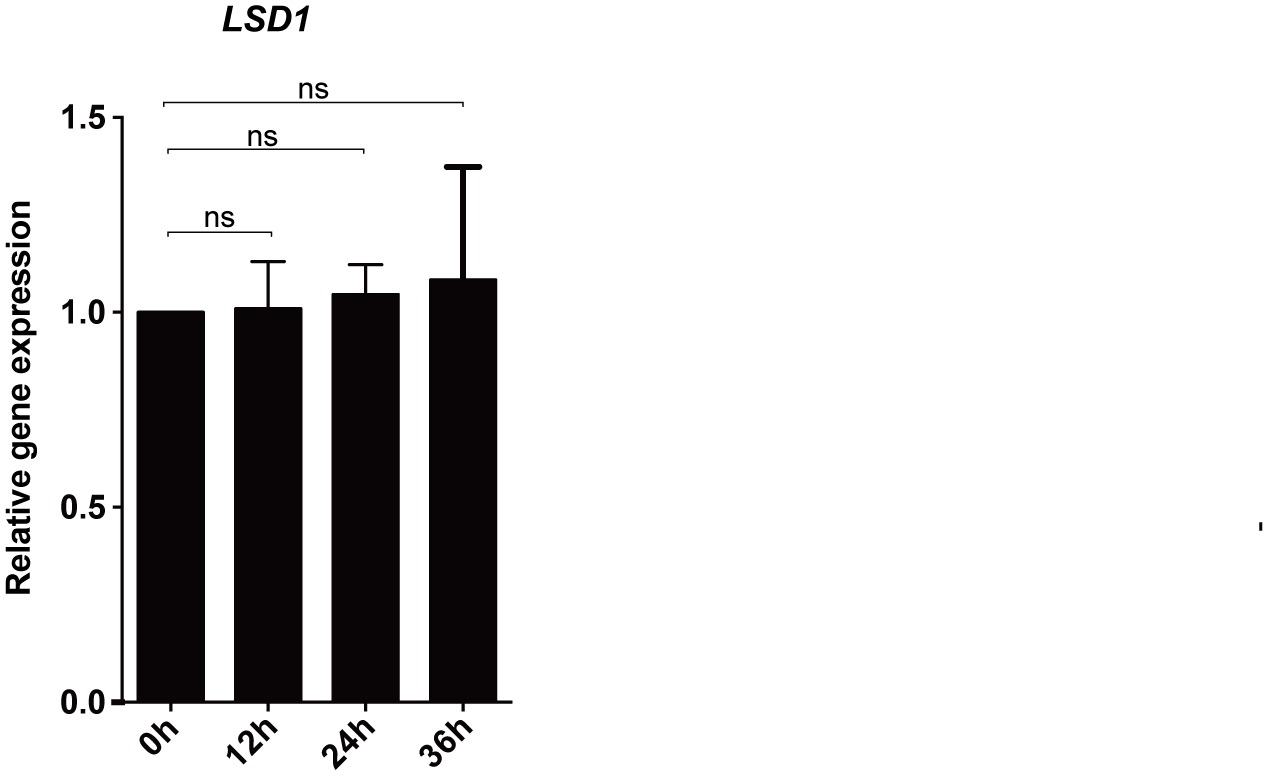

Supplement: S5 Fig — HEK293T cells grown in six-well plates were treated with IFNα (200U/ml) for the indicated time periods and were then collected following by qPCR analyses of the mRNA levels of LSD1. The data are shown as mean + s.d. of three independent experiments. ns, p >0.05. (TIF) [file ppat.1006773.s005.tif]
